# The E‐cadherin‐Wnt‐mir‐994 Axis Repurposes a Cadherin Switch for Niche Robustness and Germline Stem Cell Maintenance

**DOI:** 10.1111/cpr.70245

**Published:** 2026-06-26

**Authors:** Renjun Tu, Hiu Laam Yau, Runzhi Deng, Sarah E. Webb, Shiyuan Chen, Jianquan Ni, Ting Xie

**Affiliations:** ^1^ Division of Life Science The Hong Kong University of Science and Technology, Clear Water Bay, Kowloon, Hong Kong Special Administrative Region (SAR) Hong Kong China; ^2^ School of Life Science and Technology, The Key Laboratory of Developmental Genes and Human Disease, Southeast University Nanjing China; ^3^ Stowers Institute for Medical Research Kansas City Missouri USA; ^4^ Department of Basic Medical Sciences School of Medicine, Tsinghua University Beijing China

## Abstract

The resilience and robustness of the stem cell niche are critical for long‐term tissue homeostasis, yet the molecular circuits that ensure this stability remain poorly understood. In the *Drosophila* ovarian germline stem cell niche, we investigate this fundamental question through the lens of adhesion, focusing on the role of N‐cadherin in the somatic inner germarial sheath (IGS) cells. While the specific loss of N‐cadherin alone is inconsequential, we discover that it becomes essential upon the loss of E‐cadherin, revealing a critical, context‐dependent function. This functional interplay is governed by a precise molecular circuit wherein E‐cadherin cell‐autonomously represses N‐cadherin expression via a linear Wnt‐*mir‐994* signalling axis. Strikingly, this regulatory relationship constitutes a cadherin switch, which is repurposed within the niche not to promote dispersal, but to enforce resilience. The E‐cadherin‐to‐N‐cadherin switch acts as a vital compensatory mechanism: the ectopic upregulation of N‐cadherin upon E‐cadherin depletion is essential to maintain IGS cell survival and their long cellular processes, thereby rescuing niche integrity and preventing GSC loss. Our study defines the function for N‐cadherin in IGS cells, unveils the E‐cadherin‐Wnt‐*mir*‐*994*‐N‐cadherin axis and demonstrates the repurposing of a classic developmental module as a robustness circuit to safeguard the stem cell niche. This repurposed cadherin switch reveals an axis for targeting the resilience of niche‐stem cell interplay, and also informs new strategies for stabilizing niche environments in regenerative medicine or targeting the resilient cancer stem cell microenvironment.

## Graphical abstract

In the *Drosophila* ovarian niche, an E‐cadherin‐to‐N‐cadherin switch, mediated by Wnt‐*mir‐994* signalling, is repurposed to ensure niche resilience. This compensatory mechanism maintains niche integrity and stem cell support upon E‐cadherin loss, revealing a robustness circuit.

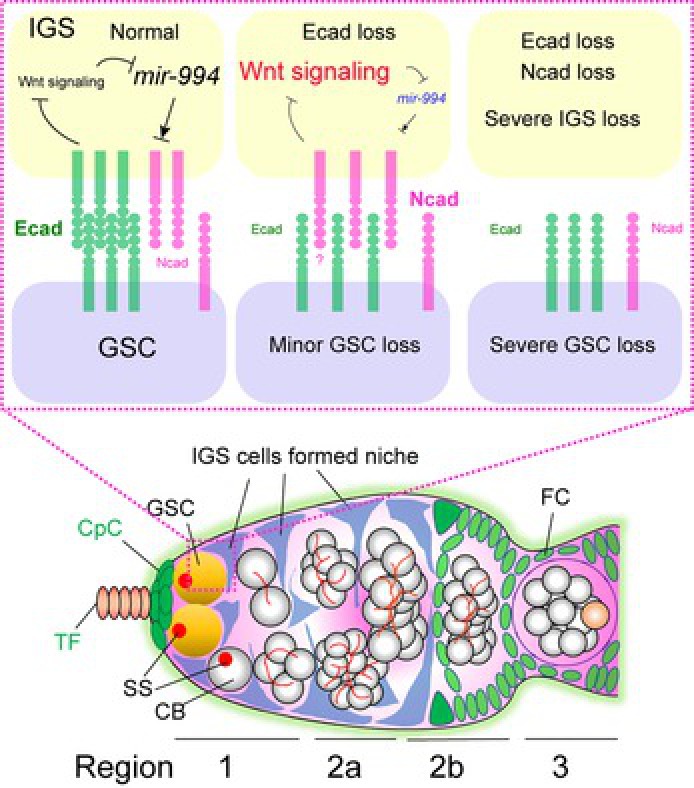

## 1 Introduction

Stem cells maintain tissue homeostasis through their dual capacity for self‐renewal and differentiation, a balance exquisitely regulated by intrinsic factors and their specialized microenvironment—the stem cell niche [1, 2]. During development, niche‐derived cues direct stem cell differentiation and orchestrate organogenesis [3]. In adults, the niche dynamically controls stem cell fate to sustain tissue repair and regeneration [4]. Niche dysfunction contributes to both degenerative diseases, through failed stem cell support, and hyperproliferative disorders, including cancer [5]. Malignant cells can co‐opt normal niche components to create a ‘tumour niche’ that sustains cancer stem cells, promotes metastasis and confers therapy resistance [5]. Therapeutically, niche manipulation offers promising strategies: rejuvenating damaged niches could enhance regeneration, while disrupting the tumour niche may overcome treatment resistance and prevent relapse, shifting the therapeutic paradigm from targeting cancer cells alone to targeting their microenvironment [6].

**FIGURE 1:**
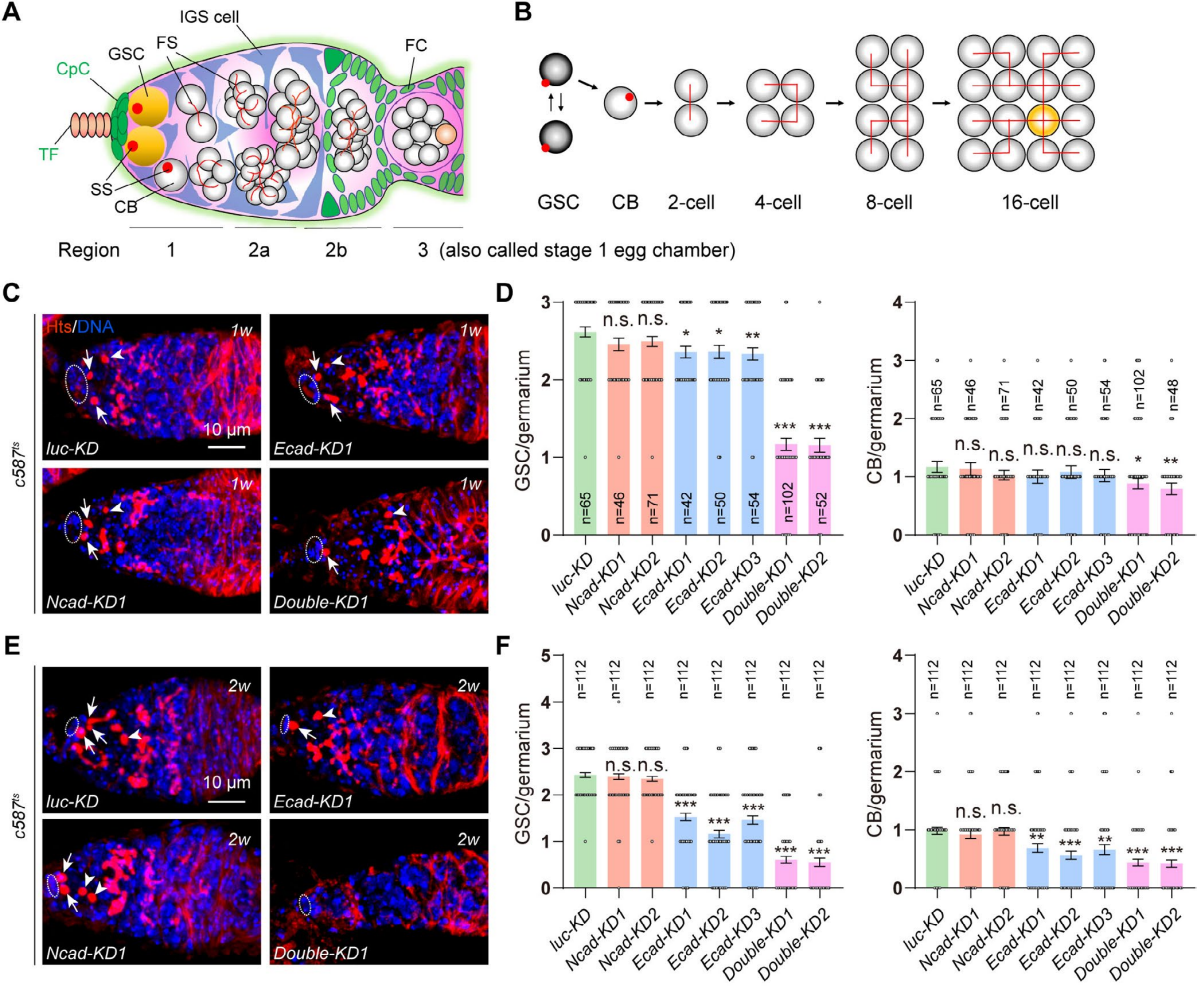
N‐cadherin compensates for E‐cadherin function in E‐cadherin knocked down IGS cells to promote GSC self‐renewal. (A) A schematic diagram of the *Drosophila* germarium, divided into three regions according to germ cell developmental stages. Region 1 contains mitotic germ cells, including germline stem cells (GSCs), cystoblasts (CBs) and mitotic cysts (2‐, 4‐ and 8‐cell cysts). Region 2 comprises ball‐like 16‐cell cysts enveloped by inner germarial sheath (IGS) cells (2a) and lens‐shaped 16‐cell cysts surrounded by follicle cells (2b). Region 3 contains a stage 1 egg chamber. Abbreviations: TF, terminal filament; CPC, cap cell; IGS, inner germarial sheath; CB, cystoblast; SS, spectrosome; FS, fusome; FC, follicle cell. (B) A GSC undergoes self‐renewing division, producing one GSC and one cystoblast (CB). The CB then undergoes four rounds of division with incomplete cytokinesis to form a 16‐cell cyst. Distinct fusome morphologies—such as those in 2‐, 4‐, 8‐ and 16‐cell cysts—allow clear identification of each developmental stage. (C–F) The one‐week (C, D) or two‐week (E, F) IGS‐specific knockdown of N‐cadherin (*Ncad‐KD*), E‐cadherin (*Ecad‐KD*) or both (*Double‐KD*) resulted in unchanged, slightly reduced and highly reduced germline stem cell (GSC) numbers, respectively, when compared with the luciferase knockdown (*luc‐KD*) control. Knockdown was conducted at 29°C. *n* = number of germaria. Scale bars, 10 μm. Student's *t*‐test: *p ≤ 0.05, **p ≤ 0.01, ***p ≤ 0.001, n.s., no significance.

The intricate interplay between stem cells and their niche has been powerfully elucidated using *Drosophila melanogaster* as a model system [7, 8]. The fruit fly has served as a pioneering model for discovering conserved principles of stem cell biology, owing to its unparalleled genetic tools, well‐defined stem cell lineages and physiological relevance [9]. In the *Drosophila* ovary, a sophisticated regulatory framework has been established, featuring a ‘self‐renewal niche’ at the germarial tip, which is composed of cap cells and anterior inner germarial sheath (IGS) cells, and a posterior ‘differentiation niche’ formed by posterior IGS cells that orchestrate the stepwise development of germline cysts (Figure 1A) [10, 11, 12, 13, 14, 15]. Cap cells anchor GSCs via E‐cadherin (encoded by *shotgun* [*shg*])‐mediated cell adhesion and secrete Decapentaplegic (Dpp) (encoded by *dpp*), a BMP family ligand, to control GSC self‐renewal by repressing their differentiation [16, 17, 18, 19]. The anterior IGS cells also contribute to GSC maintenance by expressing low levels of E‐cadherin [20]. When a GSC divides, it generates one self‐renewing GSC, which remains anchored to the cap cells, and one differentiating daughter cell called a cystoblast (CB). The CB divides synchronously a further four times with incomplete cytokinesis to form a 16‐cell cyst via mitotic 2‐cell, 4‐cell, and 8‐cell cyst intermediates (Figure 1B) [21]. The posterior IGS cells extend their long cellular processes over the CBs, mitotic cysts and early 16‐cell cysts to form multiple compartments for controlling the stepwise differentiation of the GSC progeny [10]. While the mechanisms by which niches instruct stem cells have been extensively studied, a fundamental question regarding the molecular mechanisms that ensure the structural and functional stability of the niche itself remains largely unexplored. The survival of key niche cells, such as IGS cells, is known to depend on interactions with germ cells [11, 22], yet the specific molecular mediators safeguarding this delicate interdependence are poorly defined.

Cell adhesion molecules are prime candidates for maintaining niche architecture and signalling [23]. Among them, the classic cadherins, E‐cadherin and N‐cadherin, mediate homophilic adhesion and contact‐dependent signalling, playing critical roles in tissue integrity and cell fate [24]. A quintessential example of their dynamic regulation is the epithelial‐to‐mesenchymal transition (EMT), a process fundamental to development and disease [25]. During EMT, cells lose E‐cadherin expression and subsequently upregulate N‐cadherin, a phenomenon known as the ‘cadherin switch’, which promotes cell motility and invasiveness [26]. This switch is often triggered by the activation of Wnt signalling upon loss of E‐cadherin, leading to the induction of transcription factors like Snail and the repression of E‐cadherin itself [27]. However, whether this canonical pathway operates in stem cell niches, and to what biological end, is unknown.

Wnt signalling is a highly conserved cell–cell communication pathway that plays critical roles in development, tissue homeostasis and stem cell regulation. The canonical Wnt pathway is initiated by the binding of secreted Wnt ligands (including Wnt2, Wnt4 and Wnt6 in *Drosophila*) to Frizzled receptors and LRP5/6 co‐receptors on the receiving cell membrane [28]. This interaction stabilizes cytoplasmic *β*‐catenin (encoded by *armadillo* in *Drosophila*), which subsequently translocates into the nucleus to activate target gene expression via TCF/LEF (encoded by *pangolin* [*pan*] in *Drosophila*) transcription factors. Wnt signalling is highly active in IGS cells and has been implicated in GSC maintenance and GSC progeny differentiation [20, 29, 30, 31].

In the *Drosophila* ovarian niche, E‐cadherin is recognized for its role in anchoring germline stem cells (GSCs) to cap cells [16, 17]. Strikingly, both E‐ and N‐cadherin (encoded by *CadN* in *Drosophila*) are also expressed in the somatic IGS cells, a critical component of both the self‐renewal and differentiation niches. However, the function of N‐cadherin in IGS cells has not been well investigated, and the potential regulatory interplay between these two cadherins within the niche remains a complete mystery. This gap in knowledge raises compelling questions: Do E‐ and N‐cadherin function independently, antagonistically or cooperatively in this context?

Here, we address these questions by uncovering a novel, non‐canonical function of the cadherin switch in ensuring niche resilience. We demonstrate that in IGS cells, E‐cadherin constitutively represses N‐cadherin expression via a linear signalling axis involving Wnt and the microRNA *mir‐994*. This repression is not merely inhibitory but establishes a critical fail‐safe mechanism: upon loss of E‐cadherin, the consequent derepression of N‐cadherin becomes essential for IGS cell survival and function, thereby preventing GSC loss. Our findings reveal the E‐cadherin‐Wnt‐*mir‐994*‐N‐cadherin axis, a previously unidentified regulatory circuit to build robustness into the very foundation of the stem cell niche. This work fundamentally expands our understanding of how niches are maintained and offers a new paradigm for considering cellular resilience in regenerative and disease contexts.

## 2 Materials and Methods

### 2.1 Drosophila Culture

The following *Drosophila* stocks used in this study are described in FlyBase, unless otherwise specified: *bab1‐Gal4* [32], *c587‐Gal4*, *hsFlp*, *Act > stop > Gal4*, *UAS‐mCD8‐GFP, UAS‐GFP, tubulin‐gal80* ^*ts*^, *luc* RNAi (BL31603), *UAS‐luc* (BL64774), *Ncad* RNAi (BL27503 and TH00686), *Ecad* RNAi (BL27689, TH00414, BL32904), *Ncad* and *Ecad* double RNAi (TH12081, TH00686 + BL27689), *Dcr‐1* RNAi (BL28598, BL34826, BL42901), *mir‐994* sponge (BL61478), *UAS‐mir‐994* (BL60650), *dsh* RNAi (BL31306), *pan* RNAi (BL40848), *UAS‐Myc‐Arm* ^*S10*^ (BL4782), *UAS‐Ncad* [33], *UASp‐Flag‐Ecad* and *fz3‐RFP*. The stocks prefixed with ‘BL’ were obtained from the Bloomington *Drosophila* Stock Center, whereas those prefixed with ‘TH’ were gifts from the Tsing Hua Fly Center (THFC). The flies were maintained and crossed at room temperature on standard cornmeal/molasses/agar media unless otherwise specified. For maximizing the effect of RNAi‐mediated knockdown or gene overexpression, newly eclosed flies were shifted to 29°C for a specified number of days before the analysis of ovarian phenotypes.

### 2.2 Construction of Transgenic *Drosophila* Strains

To generate the *mir‐994* ^*WT*^‐GFP transgene, a 2500‐bp genomic region, spanning from ‘tatgataggatatgttgaaa’ to ‘taattagataccaagaataa’ of *mir‐994*, was cloned into the *pGreenRabbit* (pGR) vector [34]. In the *mir‐994* ^*MU*^‐GFP construct, the predicted binding site within the 2500 bp region was randomly mutated from ‘ggatgaaaaggtccttcggtttca’ to ‘ggacagatcgagatctgcatgcat’. The constructs were inserted into the attp40 site on the second chromosome using PhiC31 integrase‐mediated transgenesis by WellGenetics Inc. (Taiwan).

### 2.3 Immunostaining and Confocal Imaging

*Drosophila* germaria immunostaining was performed as previously described [28, 35]. The following antibodies were used: mouse monoclonal anti‐Hts (1B1, DSHB; used at a 1:50 dilution), rat monoclonal anti‐Ncad (DN‐Ex, DSHB; diluted 1:20), rat monoclonal anti‐Ecad (DCAD2, DSHB; diluted 1:20), mouse monoclonal anti‐Vasa (anti‐vasa, DSHB; used at 1:50), chicken polyclonal anti‐GFP (#A10262, Invitrogen, used at 1:500), goat polyclonal anti‐GFP (#600‐101‐215, Rockland, used at 1:500), rabbit polyclonal anti‐*β*‐galactosidase (LacZ) (#08559761, MP Biomedical; at 1:500), rabbit polyclonal anti‐RFP (#600‐401‐379, Rockland; at 1:1000) and rabbit monoclonal anti‐Smad3 (phosphor S423 + S425) antibody (ab52903, Abcam; used at 1:500). Images were acquired with a Leica TCS SP5 or SP8 confocal microscope. Fluorescence intensities for the highlighted areas of interest were quantified using the Leica software or ImageJ, and the mean values of fluorescence intensities and internal controls were collected after subtraction of the background fluorescence [28, 36].

### 2.4 Fluorescence‐Activating Cell Sorting (FACS) of GFP‐Positive IGS Cells and RNA Seq

Newly eclosed flies expressing UAS‐GFP and E‐cadherin RNAi driven by *c587* ^*ts*^ in IGS cells were cultured for 1 week at 29°C. The ovaries were then dissected, placed in Grace's medium (Sigma‐Aldrich; G9771), and washed twice with 1X Dulbecco's Phosphate‐Buffered Saline (DPBS) before being centrifuged at 700g for 1 min. The ovaries were then incubated in a prewarmed collagenase solution (50D11833; Worthington) in a 15 mL conical tube in a 37°C water bath for 3 min with gentle shaking, after which the enzyme reaction was stopped by adding cold 1X DPBS + 2% FBS. The dissociated samples were washed with 1X DPBS, and then they were centrifuged at 700*g* and 4°C for 5 min, after which the cell pellet was resuspended in 1X DPBS. The cells were filtered using a 70 μm cell strainer, pelleted via centrifuged again and then resuspended in 200 μL 1X DPBS for immediate sorting of GFP‐positive cells.

Subsequently, mRNA isolation and RNA‐seq were conducted according to the published procedures [29, 37]. Briefly, 1000 cells of each of three biological replicates were harvested for control and E‐cadherin RNAi. cDNA was synthesized using SMART‐seq v4 Ultra Low Input RNA kit (634888; Takara Bio Inc.) with subsequent library preparation by Nextera XT DNA Sample Preparation kit (FC‐131‐1096; Illumina) and Index kits (FC‐131‐1002; Illumina). cDNA samples and libraries were both confirmed on a Bioanalyzer 2100 before RNAseq. Libraries were sequenced as 75‐bp high output paired reads on a NextSeq (Illumina). Each sample generated in excess of 20 million fastq counts. Raw reads were demultiplexed into Fastq format allowing up to one mismatch using Illumina bcl2fastq2 v2.18. Reads were aligned to UCSC genome dm6 with TopHat v2.0.13, default parameters, using Ensembl 87 gene models. FPKM values were generated using Cufflinks v2.2.1 with ‘‐u ‐max‐bundle‐frags 100000000’. Read counts were generated using HTSeq‐count with ‘‐m intersection‐nonempty’. RNA‐seq data that support the findings of this study have been deposited in the Gene Expression Omnibus under accession numbers GSE318190.

### 2.5 EdU and TUNEL Labeling

EdU labeling was conducted with the Click‐iT EdU Alexa Fluor 488 imaging kit (C10337, Thermo Fisher Scientific), as previously described [35]. In addition, the TUNEL labeling assay was performed using the Click‐iT Plus TUNEL assay kit for in situ apoptosis detection (C10618, Thermo Fisher Scientific). In brief, the ovaries were dissected and fixed according to standard protocols [35]. They were then washed three times with PBS containing 0.1% Triton X‐100, after which the EdU or TUNEL protocols were conducted according to the manufacturer's instructions. At the end of these protocols, the ovaries were immunolabelled with primary and secondary antibodies and co‐stained with DAPI, using standard methods.

### 2.6 Ovarian Somatic Cell Culture and dsRNA‐Mediated Knockdown

*Drosophila* ovarian somatic cell culture was conducted as described in previous studies [38, 39]. For the dsRNA‐mediated knockdown of *Ecad*, dsRNA was generated using the MEGAscript T7 Transcription kit (AM1334, Thermo Fisher Scientific) with a PCR‐amplified DNA template. The primers used to amplify the template were:

- Forward primer: taatacgactcactatagggtgactatcagcgccagtgac.
- Reverse primer: taatacgactcactatagggcgtgtgtattccgcacaatc.

### 2.7 Protein Purification and In Vitro DNA Binding Assays

GST‐Pan‐HMG was purified as previously described [28]. An in vitro DNA‐protein binding assay was performed with the LightShift Chemiluminescent EMSA Kit (20,148, Thermo Fisher Scientific). Glycerol (4.35%), magnesium chloride (5 mM), poly(dI‐dC) (50 ng/mL) and NP‐40 (0.05%) were included in the binding reaction. For each 20 μL sample, 0.1 nM biotin‐labelled probes and 10 μg of purified GST protein or GST fusion proteins were used. The 5′ biotin‐tagged double‐stranded DNA WT probe (ggatgaaaaggtccttcggtttca) and mutant probe (ggacagatcgagatctgcatgcat) used in this assay were synthesized by Beijing Tsingke Biotech Co. Ltd.

### 2.8 ChIP and qPCR

ChIP was performed with the Pierce Agarose ChIP kit (26156, Thermo Fisher Scientific), according to the manufacturer's instructions. Three hundred pairs of *c587‐Gal4* overexpressed control or *UAS‐Myc‐Arm* ^*S10*^ ovaries were dissected and digested with type II collagenase (50D11833; Worthington). Late‐stage egg chambers and mature eggs were filtered and removed, after which a ChIP grade anti‐Myc antibody (ab9132, Abcam) was used for chromatin pull‐down.

Primers targeting *Act5C* (atcgggatggtcttgattctg and actccaaacttccaccactc) and *mir‐994* (agatccgaatgcgagtatcc and caaagcccagtgatgcttga) were used for qPCR. For validation of dsRNA knockdown efficiency of E‐cadherin, primers targeting *Rpl10* (atgctaagctgtcgcacaaatg and gttcgatccgtaaccgatgt) and *E‐cadherin* (tacgacgaatccatgtcgga and ccgactccttgtcaatcttg) were used for qPCR.

### 2.9 Fluorescent RNA In Situ Hybridization (FISH)

The hybridization chain reaction (HCR) was used to achieve high sensitivity FISH. With regards to the probes, *Ncad* mRNA was purchased from Molecular Instruments Inc. (code # PRF857), whereas the probe against *mir‐994‐5p* (5′‐gaggagggcagcaaacgggaagagtcttcctttacgatattatcacggctactatttccttagatatagcattctttcttgaggagggcagcaaacgggaagag‐3′) was designed according to previous reports [40, 41]. Ovaries were first immunolabelled with anti‐Vasa antibody according to established protocols [10, 42]. Then, the HCR v3.0 method was applied to perform in situ hybridization for germaria imaging [40].

### 2.10 S2 Cell Culture and Transfection

S2 cells were grown at 25°C in HyClone SFX‐insect cell culture media (SH30278.02, Cytiva). Transfections were performed using the X‐treme GENE HP (6366546001, Roche) transfection reagent, according to the manufacturer's instructions. To generate the *UASz‐Ncad‐EGFP* construct, the predicted target sequence of *Ncad* (cgcatcacagtgaaatgtccttggtcc) by *mir‐994* was fused at the N‐terminus of the EGFP coding sequence. For the overexpression of *mir‐994* in S2 cells, the sequences between ‘tgaagaaacccagcagcggg’ and ‘aagtaggttggtggataaatatattg’ were cloned into the *UASz* vector, resulting in the *UASz‐mir‐994* construct. The expression of *UASz‐Ncad‐EGFP* and *UASz‐mir‐994* in S2 cells was driven by pAc‐Gal4 (#24344, Addgene) [43].

### 2.11 Quantification of Fluoresce Intensity

**FIGURE 2:**
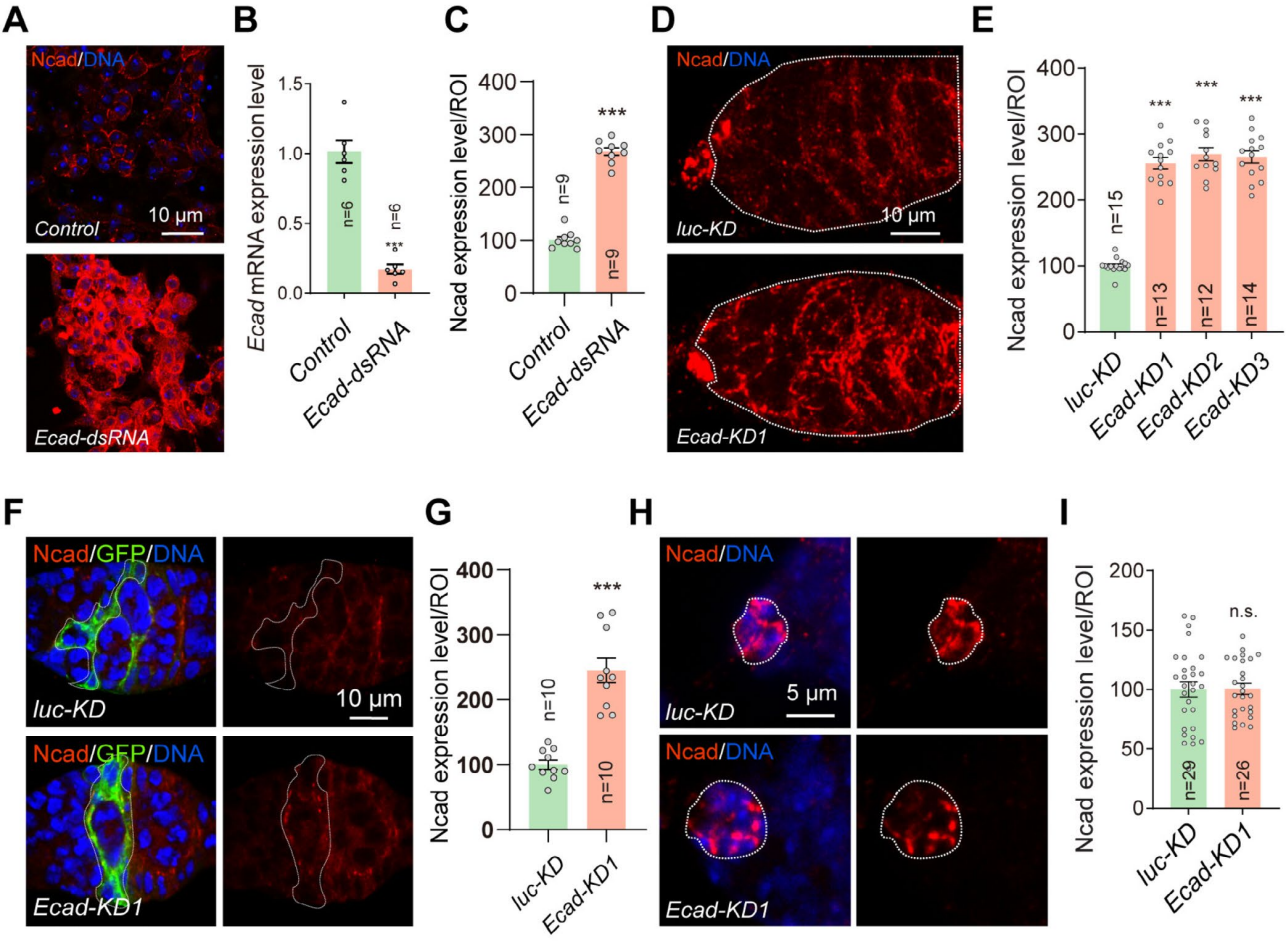
Figure N‐cadherin protein level is upregulated following E‐cadherin knockdown in IGS cells. (A–C) The *dsRNA*‐mediated knockdown of *Ecad* in cultured ovarian somatic cells resulted in a significant increase in the expression of Ncad. *n* = number of technical replicates (B) or number of ROIs (C). Scale bars, 10 μm. (D, E) The *c587* ^*ts*^‐mediated knockdown of *Ecad* in IGS cells significantly increased the expression of Ncad (*n* = number of ROIs). Scale bars, 10 μm. (F, G) Single optical sections (and associated quantification) demonstrate that the *c587* ^*ts*^‐mediated *Ecad‐KD* in IGS cells, which are expressing GFP, resulted in a significant increase in the expression of Ncad specifically in the IGS cells (*n* = number of ROIs). Scale bars, 10 μm. (H, I) The *bab1* ^*ts*^‐mediated knockdown of *Ecad* in IGS cells did not alter the expression of Ncad (*n* = number of ROIs). Scale bars, 5 μm. Student's *t*‐test: ***p ≤ 0.001, n.s., no significance.

Fluorescence intensity quantification was performed using a Leica SP8 confocal microscope. Regions of interest (ROIs) were manually delineated, and the mean fluorescence intensity values for all ROIs were exported for statistical analysis. For Figures S1C,E and 2H, cap cells were identified based on their positional cues within the germarium and DAPI staining intensity (characterized by condensed, brighter nuclei). The mean fluorescence intensity was recorded for these cells. For Figure 2A, the mean fluorescence intensity was measured over the entire image area where cells can be identified. For Ncad quantification in IGS cells (Figures S1, 2D, 3D and 4E,K), ROIs encompassing the germarium region excluding cap cells were selected. Although germ cells were present within these ROIs, Ncad expression in germ cells was negligible (near background levels). This approach was validated by c587‐gal4‐mediated Ncad knockdown, which abolished detectable Ncad signal in these ROIs (Figure S1). For FISH mRNA spot quantification (Figures 4C, 5B, S5C and S6C): germ cells were labelled with Vasa; consequently, Vasa‐negative cells (IGS cells) were demarcated, and their mean fluorescence intensity was recorded. For mir‐994‐GFP reporter quantification (Figure 5G,I), similarly, Vasa‐negative cells (IGS cells) were identified and analysed for mean fluorescence intensity. For quantification of immunofluorescence intensity, at least three independent biological replicates were conducted. All statistical comparisons were conducted using two‐tailed Student's *t*‐tests.

**FIGURE 3:**
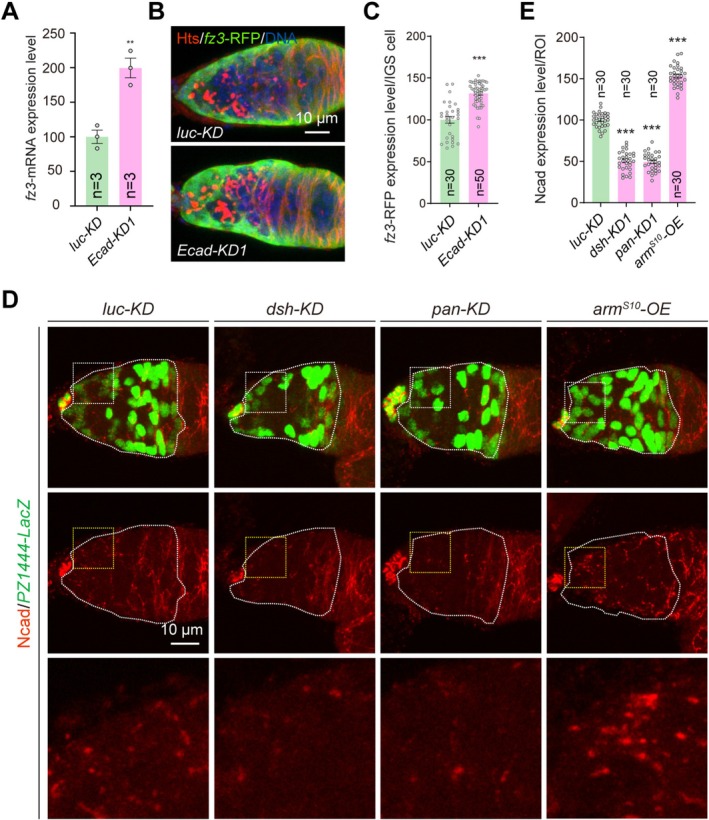
Figure Wnt signalling promotes the expression of N‐cadherin. (A) RNA‐seq indicates that the mRNA expression of *fz3*, a downstream target of Wnt signalling, is upregulated in *Ecad‐KD* IGS cells (*n* = biological replicates). (B, C) *fz3*‐RFP is significantly upregulated in *Ecad‐KD* IGS cells (*n* = IGS cells). Scale bars, 10 μm. (D, E) PZ1444 labelled IGS cells. After a brief knockdown or overexpression at 29°C (3 days), Ncad expression decreased in *dsh‐KD* and *pan‐KD* germaria but increased in *arm* ^*S10*^ *‐OE* germaria (*n* = number of ROIs). Scale bars, 10 μm. Student's *t*‐test: **p ≤ 0.01, ***p ≤ 0.001.

**FIGURE 4:**
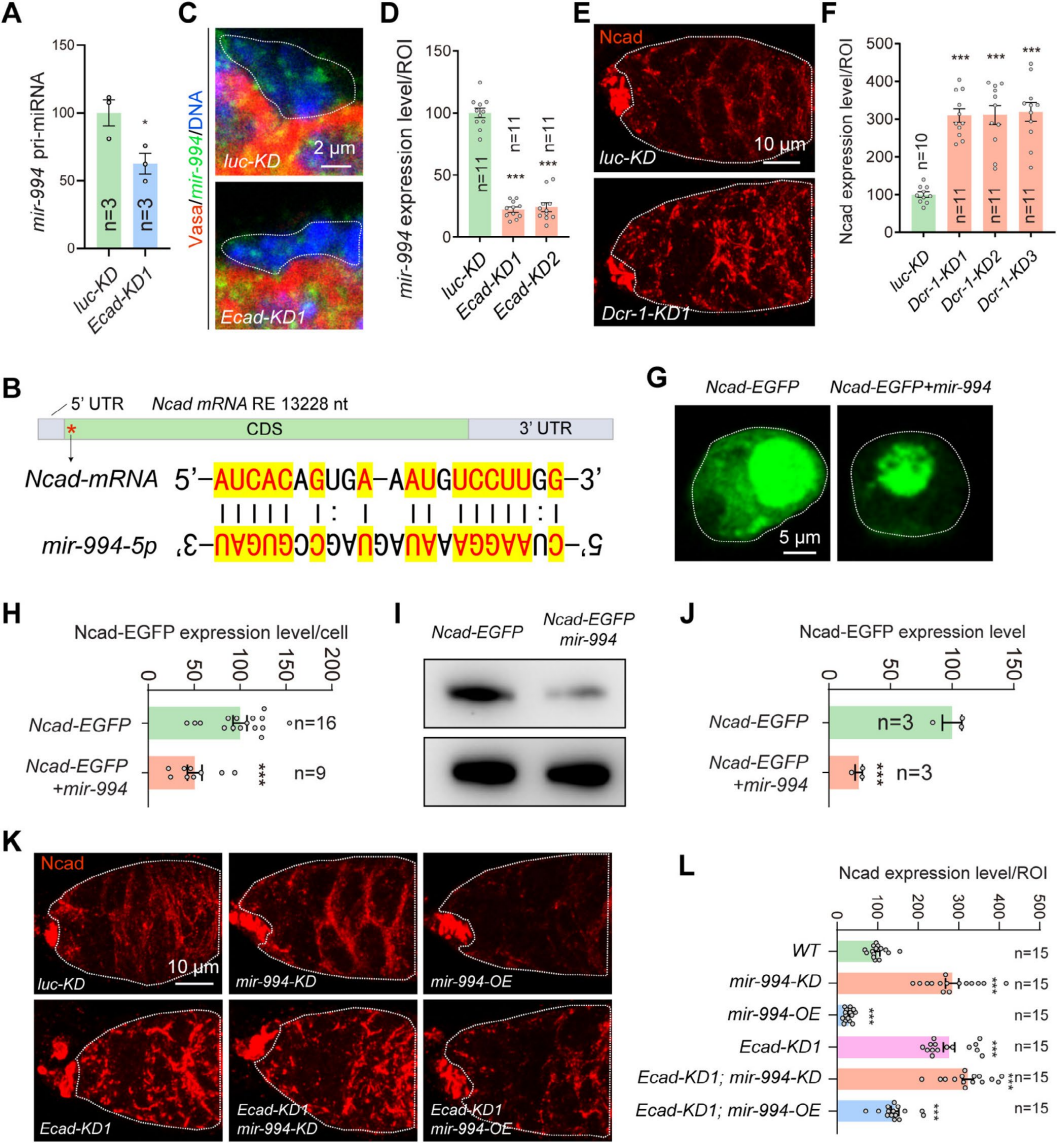
Figure *mir‐994* limits the expression of N‐cadherin (Ncad) in IGS cells. (A) RNA‐seq indicates that the expression of *mir‐994* is reduced in *Ecad‐KD* IGS cells. *n* = the number of biological replicates (*n* = technical replicates). (B) The alignment of *Ncad* mRNA and *mir‐994* sequences reveals that *mir‐994* might target ‘aucacagugaaauguccuugg’ in *Ncad* mRNA. (C, D) HCR‐FISH showed that the expression of *Ncad* mRNA was comparable in both *luc‐KD* and *Ecad‐KD* IGS cells (Vasa‐negative as shown by region bounded by white line) (*n* = number of ROIs). Scale bars, 2 μm. (E, F) The *c587* ^*ts*^‐mediated knockdown of *Dcr‐1* in IGS cells significantly increased the expression of Ncad (*n* = number of ROIs). Scale bars, 10 μm. (G–J) The immunolabelling (G) and immunoblotting (I) results demonstrated that overexpressing *mir‐994* in S2 cells resulted in a reduction in the expression of EGFP, which was fused to the predicted target site of *mir‐994* in the *Ncad* mRNA. *n* = number of cells (H) or number of technical replicates (J). Scale bars, 5 μm. (K, L) The immunolabelling results indicated that in *luc‐KD* and *Ecad‐KD* germaria, the expression of Ncad was significantly increased and decreased when *mir‐994* was knocked down or overexpressed, respectively (*n* = number of ROIs). Scale bars, 10 μm. Student's *t*‐test: *p ≤ 0.05, ***p ≤ 0.001.

**FIGURE 5:**
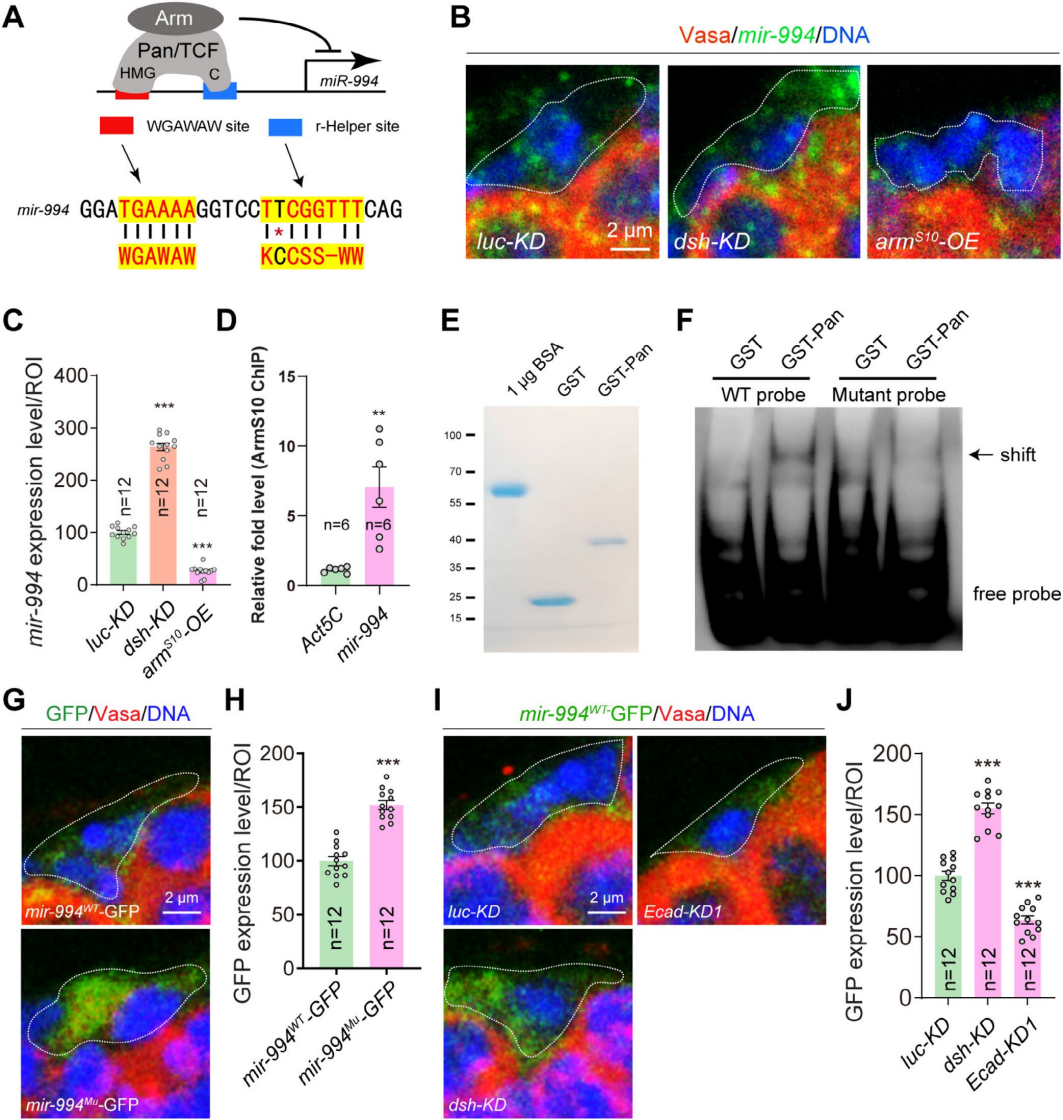
Figure Wnt signalling directly repressed the expression of *mir‐994*. (A) The *mir‐994* genome contains the conserved WGAWAW (*W* = *A* or *T*) and *r*‐Helper sites, which are known to be involved in Wnt signalling‐mediated gene repression. (B, C) The HCR‐FISH showed that the expression of *mir‐994* was increased in *dsh‐KD* and reduced in *arm* ^*S10*^ *‐OE* IGS cells (Vasa‐negative, region bounded by white line) (*n* = number of ROIs). Scale bars, 2 μm. (D) ChIP followed by qPCR indicated that the active form of Arm can bind to the *mir‐994* genome in IGS cells. (E, F) The EMSA demonstrated that purified GST‐Pan‐HMG proteins can bind to the WGAWAW and *r*‐Helper sites of *mir‐994*. However, when mutations are introduced at these sites, then GST‐Pan‐HMG protein *mir‐994* binding is abolished. (G, H) Mutations introduced in the WGAWAW and r‐Helper sites of *mir‐994* led to an upregulation of *mir‐994* ^*MU*^‐GFP but not *mir‐994* ^*WT*^‐GFP in IGS cells (*n* = number of ROIs). Scale bars, 2 μm. (I, J) In IGS cells, the expression of *mir‐994* ^*WT*^‐GFP increased when *dsh* was knocked down and decreased when *Ecad* was knocked down (*n* = number of ROIs). Scale bars, 2 μm. Student's *t*‐test: **p ≤ 0.01, ***p ≤ 0.001.

### 2.12 Quantification of GSCs, CBs IGS Cells and Statistical Analysis

GSCs, CBs and IGS cells were quantified according to our previous studies [44]. In brief, spectrosome‐containing single germ cells that were attached to cap cells were considered to be GSCs, whereas those that were not attached to the cap cells were identified as CBs. These were identified and quantified by fluorescence microscopy. IGS cells were quantified using the PZ1444‐LacZ reporter such that the enhancer trap line PZ1444 expressing nuclear LacZ was used to identify both IGS and cap cells. These cell types were then distinguished based on differences in their location and size of nucleus. For example, cap cells are smaller and rounder than IGS cells, and they cluster together at the anterior tip of the germaria. For quantification of GSC, CB and IGS cell numbers, at least two independent biological replicates were performed for each experiment.

Statistical analysis was conducted using Microsoft Excel or GraphPad Prism 9. The raw data for all statistical graphs are provided in Table S1. Data are presented as the mean or mean ± SEM and compared using the Student's *t*‐test. *p* ≤ 0.05 was considered to be statistically significantly different and asterisks were used to identify the level of significance such that ***p ≤ 0.001, **p ≤ 0.01 and *p ≤ 0.05. Data that were not significantly different were labelled ‘n.s.’

## 3 Results

### 3.1 E‐ and N‐cadherin Function Cooperatively in IGS Cells to Maintain Germline Stem Cells

In the adult *Drosophila* germarium, GSCs are maintained by two niches composed of terminal filament, cap cells and inner germarial sheath (IGS) cells (Figure 1A,B). Cap cells and the anterior‐most IGS cells directly contact GSCs and constitute the self‐renewal niche, while the remaining IGS cells interact with early GSC progeny to form the differentiation‐promoting niche [11]. Although E‐cadherin is well established for its role in anchoring GSCs to cap cells [16], we found that both E‐ and N‐cadherin are also expressed in the somatic IGS cells, albeit at lower levels when compared with their expression in cap cells (Figure S1).

To investigate their functional roles, we used the Gal4‐UAS system to perform shRNA‐mediated knockdown of E‐ or N‐cadherin. The efficiency of each RNAi line was validated prior to functional analysis (Figure S1). To achieve IGS‐specific knockdown in adult flies, we employed the *c587‐Gal4* driver combined with *tub‐Gal80* ^*ts*^ (*c587* ^*ts*^). Knockdown of firefly luciferase (*luc‐KD*), a gene absent in the *Drosophila* genome, served as the control.

Ovaries from both control and knockdown groups were immunolabelled with an anti‐Hts antibody to identify GSCs and CBs. At the permissive temperature (21°C), where Gal80 is functional and suppresses shRNA expression, ovaries developed normally and contained expected numbers of GSCs and CBs, confirming no leaky expression (Figure S2A,B). After shifting to the restrictive temperature (29°C) to inactivate Gal80 and induce knockdown, *Ncad‐KD* germaria exhibited no obvious changes in the numbers of GSCs and CBs compared to the *luc‐KD* control, even after 1 or 2 weeks (Figure 1C–F), suggesting that N‐cadherin is not essential in IGS cells for GSC maintenance or CB differentiation. In contrast, *Ecad‐KD* resulted in a slight loss of GSCs after 1 week, which became more severe after 2 weeks (Figure 1C–F), consistent with previous reports of E‐cadherin's requirement in IGS cells [20]. Strikingly, simultaneous knockdown of both E‐ and N‐cadherin led to a more severe reduction in GSC and CB numbers than *Ecad‐KD* alone (Figure 1C–F). The decrease in CBs is likely a secondary consequence of GSC loss.

### 3.2 E‐cadherin Depletion in IGS Cells Triggers N‐cadherin Protein Upregulation

The dispensability of N‐cadherin in wild‐type IGS cells made it difficult to explain why its depletion exacerbated GSC loss upon E‐cadherin knockdown. To resolve this paradox, we hypothesized that E‐cadherin depletion might upregulate N‐cadherin expression. To test this, we first utilized an established *Drosophila* ovarian somatic cell culture system derived from *bag of marbles* (*bam*) mutant ovaries [39], which supports the growth and maintenance of GSCs through soma–germline interactions and retains key features of the in vivo niche. In this system, double‐stranded RNA‐mediated knockdown of E‐cadherin in cultured ovarian somatic cells significantly increased N‐cadherin levels (Figure 2A–C). Similarly, *c587* ^*ts*^‐mediated E‐cadherin knockdown in adult IGS cells led to marked N‐cadherin upregulation (Figure 2D,E). This effect was further confirmed in GFP‐labelled IGS cells following E‐cadherin depletion (Figure 2F,G). Interestingly, knocking down E‐cadherin in cap cells with bab1‐Gal4 did not affect N‐cadherin expression level (Figure 2H,I). These findings indicate that the enhanced GSC loss upon double knockdown results from ablation of the compensatory N‐cadherin protein that is upregulated upon E‐cadherin loss, which is consistent with previous reports of functional compensation between cadherins [45, 46].

In contrast, N‐cadherin knockdown did not affect E‐cadherin expression (Figure S3A,B). Moreover, N‐cadherin overexpression in IGS cells did not alter GSC or CB numbers, indicating that elevated N‐cadherin alone does not perturb self‐renewal or differentiation (Figure S4A,B). Importantly, IGS‐specific overexpression of E‐cadherin alone or together with N‐cadherin partially rescued the GSC loss caused by double knockdown (Figure S4C,D), confirming that both cadherins cooperate to maintain GSCs.

### 3.3 E‐cadherin Represses N‐cadherin Expression via Wnt Signalling

The upregulation of N‐cadherin following E‐cadherin loss suggested a specific regulatory relationship. To investigate the mechanism, we performed RNA‐seq on FACS‐purified GFP‐labelled control and E‐cadherin knockdown IGS cells. Gene Ontology (GO) enrichment analysis revealed distinct functional signatures associated with the upregulated and downregulated gene sets. Among the genes significantly upregulated upon E‐cadherin loss, we observed a strong enrichment for terms related to cellular adhesion and neuronal development, including cell‐cell adhesion (19 genes), neurogenesis (39 genes), neuron differentiation (35 genes) and neuron development (29 genes). In contrast, the downregulated genes were predominantly enriched for terms associated with transport and ion channel activity, such as ‘transmembrane transport’ (39 genes), ‘metal ion transport’ (18 genes), ‘regulation of cation channel activity’ (7 genes) and ‘regulation of transporter activity’ (7 genes) (Figure S5A). Surprisingly, E‐cadherin knockdown did not alter N‐cadherin mRNA levels (Figure S5B), a result confirmed by hybridization chain reaction RNA fluorescence in situ hybridization (HCR‐FISH) (Figure S5C,D). These data indicated that E‐cadherin represses N‐cadherin protein expression through a post‐transcriptional mechanism.

Notably, RNA‐seq revealed significant upregulation of *fz3*, a Wnt signalling reporter, in E‐cadherin‐deficient IGS cells (Figure 3A). Consistent with previous work in imaginal disc epithelia [47], loss of E‐cadherin in IGS cells activated Wnt signalling, as demonstrated using an *fz3*‐RFP reporter (Figure 3B,C). Thus, E‐cadherin loss activates Wnt signalling without affecting N‐cadherin transcript levels.

We next asked whether Wnt signalling regulates N‐cadherin expression. Knockdown of the Wnt transducers Dsh (encoded by *dishevelled* [dsh]) or Pan (encoded by *pangolin* [pan]) significantly reduced N‐cadherin protein in IGS cells after 3 days, whereas overexpression of constitutively active Armadillo (Arm^S10^) increased it (Figure 3D,E). These results confirm that Wnt signalling promotes N‐cadherin protein expression in IGS cells.

### 3.4 *mir‐994* Represses N‐cadherin Protein Translation in IGS Cells

Since miRNAs often regulate gene expression post‐transcriptionally, we analysed our RNA‐seq data and found that *pri‐mir‐994* and *pri‐mir‐986* were significantly downregulated upon E‐cadherin knockdown (Figure S6A,B). *mir‐994* was predicted to target a site near the translation start region of N‐cadherin mRNA (Figure 4A,B). RNA FISH confirmed that mature *mir‐994* was downregulated in *Ecad‐KD* IGS cells (Figure 4C,D). Knockdown of Dicer‐1 (Dcr‐1), an endoribonuclease which functions in microRNA‐ (miRNA) gene silencing through cleaving hairpin precursor miRNAs (pre‐miRNA) to generate mature miRNAs (miRNAs), increased N‐cadherin protein (Figure 4E,F), supporting miRNA involvement in N‐cadherin repression.

We tested whether *mir‐994* directly represses N‐cadherin using an Ncad‐EGFP reporter containing the *mir‐994* target site. Co‐expression of *mir‐994* with this reporter in S2 cells significantly reduced EGFP levels by immunostaining and western blot (Figure 4G–J). In IGS cells, *mir‐994* knockdown increased N‐cadherin protein, while its overexpression reduced it (Figure 4K,L). Furthermore, *mir‐994* knockdown enhanced and its overexpression suppressed N‐cadherin upregulation in *Ecad‐KD* IGS cells (Figure 4K,L). Importantly, N‐cadherin mRNA levels were unaffected by *mir‐994* manipulation (Figure S6C,D), confirming that *mir‐994* represses N‐cadherin at the translational level.

### 3.5 Wnt Signalling Directly Represses *mir‐994* Expression

We hypothesized that Wnt signalling connects E‐cadherin to *mir‐994*. Upon the activation of Wnt signalling, Arm interacts with Pan to form a protein complex to activate or suppress target gene expression. The Arm/Pan complex can repress transcription by binding WGAWAW and r‐Helper sites [48]. We identified conserved sites of both types in the *mir‐994* genomic region (Figure 5A). HCR‐FISH showed that *pri‐mir‐994* levels increased upon Dsh knockdown and decreased upon Arm^S10^ overexpression (Figure 5B,C), indicating Wnt signalling represses *mir‐994* transcription. ChIP using Myc‐tagged Arm^S10^ confirmed Arm binding to the *mir‐994* locus (Figure 5D). Electrophoretic mobility shift assay (EMSA) revealed direct binding of the DNA binding domain (HMG) of Pan (i.e., GST‐Pan‐HMG) to the wild‐type, but not mutant, WGAWAW and r‐Helper sites (Figure 5E,F).

Finally, we cloned the wild‐type and Pan‐binding‐defective *mir‐994* promoters into the *pGreenRabbit* reporter vector [34] to generate the *mir‐994* ^*WT*^ *‐GFP* and *mir‐994* ^*mutant*^ *‐GFP* transcriptional GFP reporter lines, respectively. We found that *mir‐994* ^*mutant*^ *‐GFP* exhibited significantly higher GFP expression than *mir‐994* ^*WT*^ *‐GFP* (Figure 5G,H). Moreover, knocking down Dsh or E‐cadherin in the IGS cells also upregulated or downregulated the expression of *mir‐994* ^*WT*^ *‐GFP*, respectively (Figure 5I,J). These results demonstrate that Wnt signalling directly represses the expression of *mir‐994* in IGS cells by Arm/Pan binding to the WGAWAW and r‐Helper sites.

### 3.6 N‐cadherin Upregulation Compensates for IGS Cell Survival and Function

**FIGURE 6:**
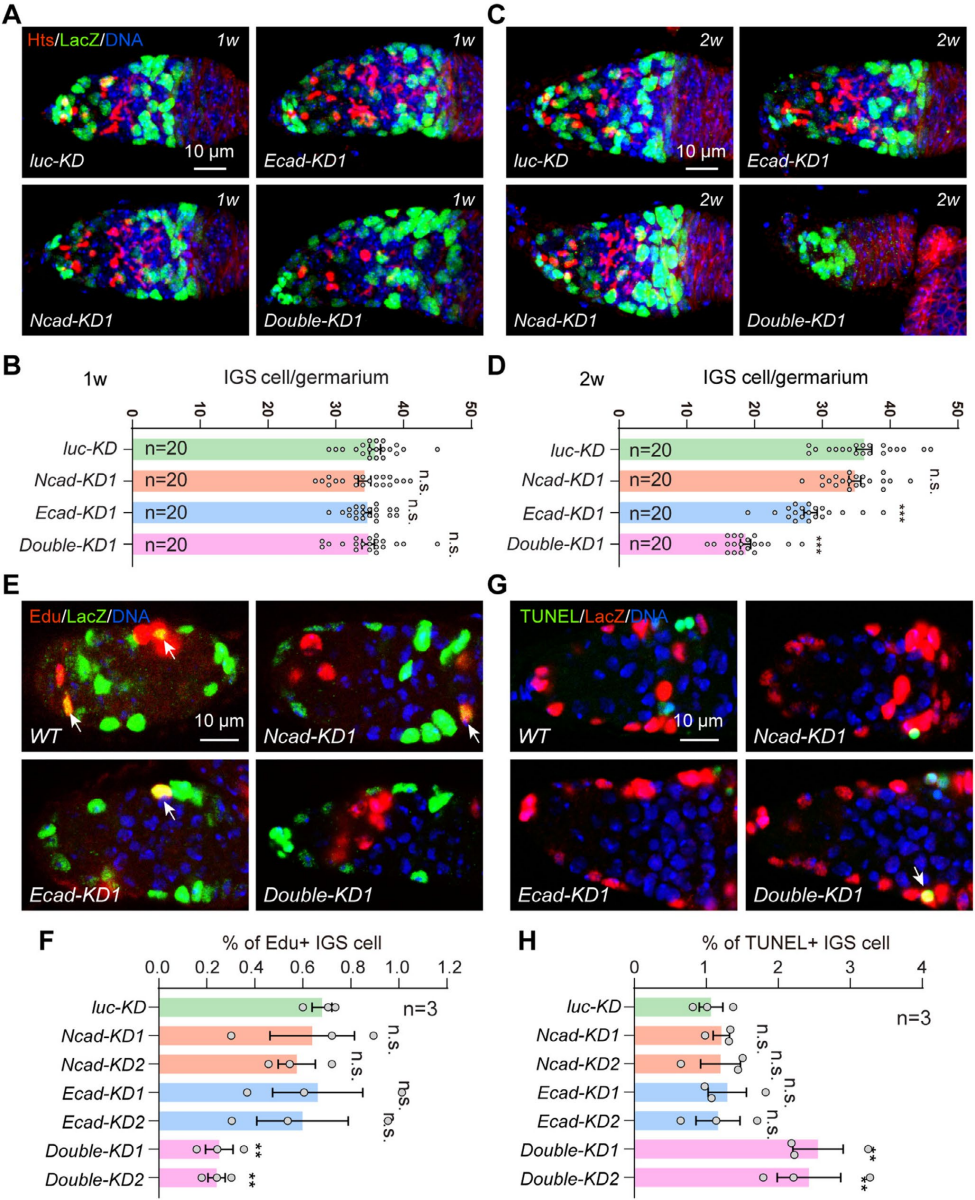
Figure E‐and N‐cadherins work cooperatively to promote IGS cell survival. (A, B) IGS‐specific *Ncad‐KD*, *Ecad‐KD* or *Double‐KD* did not affect the IGS cell number after 1 week at 29°C, when compared with the *luc‐KD* control (*n* = number of germaria). Scale bars, 10 μm. (C, D) After a 2 week knockdown at 29°C, germaria with IGS‐specific *Ncad‐KD*, *Ecad‐KD* or *Double‐KD* showed an unchanged, or slight or dramatic reduction in the number of IGS cells, respectively, when compared with *luc‐KD* control (*n* = number of germaria). Scale bars, 10 μm. (E‐H) The *c587* ^*ts*^‐mediated *Ncad‐KD* or *Ecad‐KD* did not have an obvious effect on the percentage of EdU‐positive (E, F) or TUNEL‐positive (G, H) IGS cells (arrows), whereas the *Double‐KD* clearly decreased and increased the percentage of EdU‐positive and TUNEL‐positive IGS cells, respectively (*n* = number of biological replicates). Scale bars, 10 μm. Student's *t*‐test: *p ≤ 0.05, **p ≤ 0.01, ***p ≤ 0.001, n.s., no significance.

We next asked the biological purpose of this regulatory axis. The anterior germarium appeared shrunken in double‐knockdown compared to single‐knockdown germaria (Figure 1E), suggesting IGS cell loss. To quantify the IGS cells, we used the PZ1444‐lacZ reporter [11], which expresses nuclear *β*‐galactosidase protein in both the cap and IGS cells. It is simple to distinguish between these two cell types due to their location and morphology as cap cells are small, round and in contact with the GSCs, whereas IGS cells are elongated and located lateral or posterior to the GSCs. We quantified IGS cells with PZ1444‐lacZ, and found that N‐cadherin knockdown alone did not affect IGS cell number, whereas E‐cadherin knockdown for 2 weeks significantly reduced it (Figure 6A–D). Double knockdown caused dramatically greater IGS cell loss than *Ecad‐KD* alone after 2 weeks (Figure 6A–D), demonstrating that N‐cadherin compensates for E‐cadherin in maintaining IGS cells.

Since IGS cells normally undergo slow proliferation to replenish any cells that are lost [11], the loss of these cells following double cadherin knockdown might be due to either increased apoptosis or reduced proliferation. To this end, we conducted EdU (5‐ethynyl‐2‐deoxyuridine) and TUNEL (terminal deoxynucleotidyl transferase dUTP nick end labelling) assays to examine the levels of cell proliferation and apoptosis, respectively, in IGS cells in the double knockdown germaria. We ran these assays after 1 week of knockdown since more than half of the IGS cell population was lost after 2 weeks. Compared to the control and single (*Ncad* or *Ecad*) knockdowns, the double knockdown showed a decrease in the number of EdU‐positive IGS cells and an increase in the number of TUNEL‐labelled IGS cells (Figure 6E–H). Thus, these findings indicate that E‐ and N‐cadherins have a compensatory role in regulating IGS cell proliferation and survival, thereby maintaining IGS cell homeostasis.

IGS cells extend long processes that interact with germ cells to support their self‐renewal and differentiation [11, 29, 49]. The decreased amount of cell proliferation and increased amount of apoptosis in IGS cells caused by the depletion of E‐ and N‐cadherins might therefore be due to compromised IGS‐GSC progeny interactions. To test this idea, we examined the long cellular processes of GFP‐labelled control and cadherin knockdown IGS cells, which were generated by the Ay‐Gal4 flip‐out system, as described in our previous studies [29, 50, 51]. In the individual GFP‐labelled control and single‐cadherin knockdown IGS cells, ~80% contained long cellular processes, whereas the remaining 20% had short or no cellular processes (Figure S7A,B). In contrast, among the individual GFP‐labelled double cadherin knockdown IGS cells, < 40% exhibited long cellular processes, whereas > 60% had short or no cellular processes. Therefore, the upregulation of N‐cadherin following E‐cadherin depletion serves a vital compensatory role: it promotes IGS cell survival and maintains their cellular architecture, thereby preserving the functional integrity of the niche and preventing GSC loss.

### 3.7 The E‐cadherin‐Wnt‐mir‐994‐N‐cadherin Axis Ensures Robust GSC Maintenance

**FIGURE 7:**
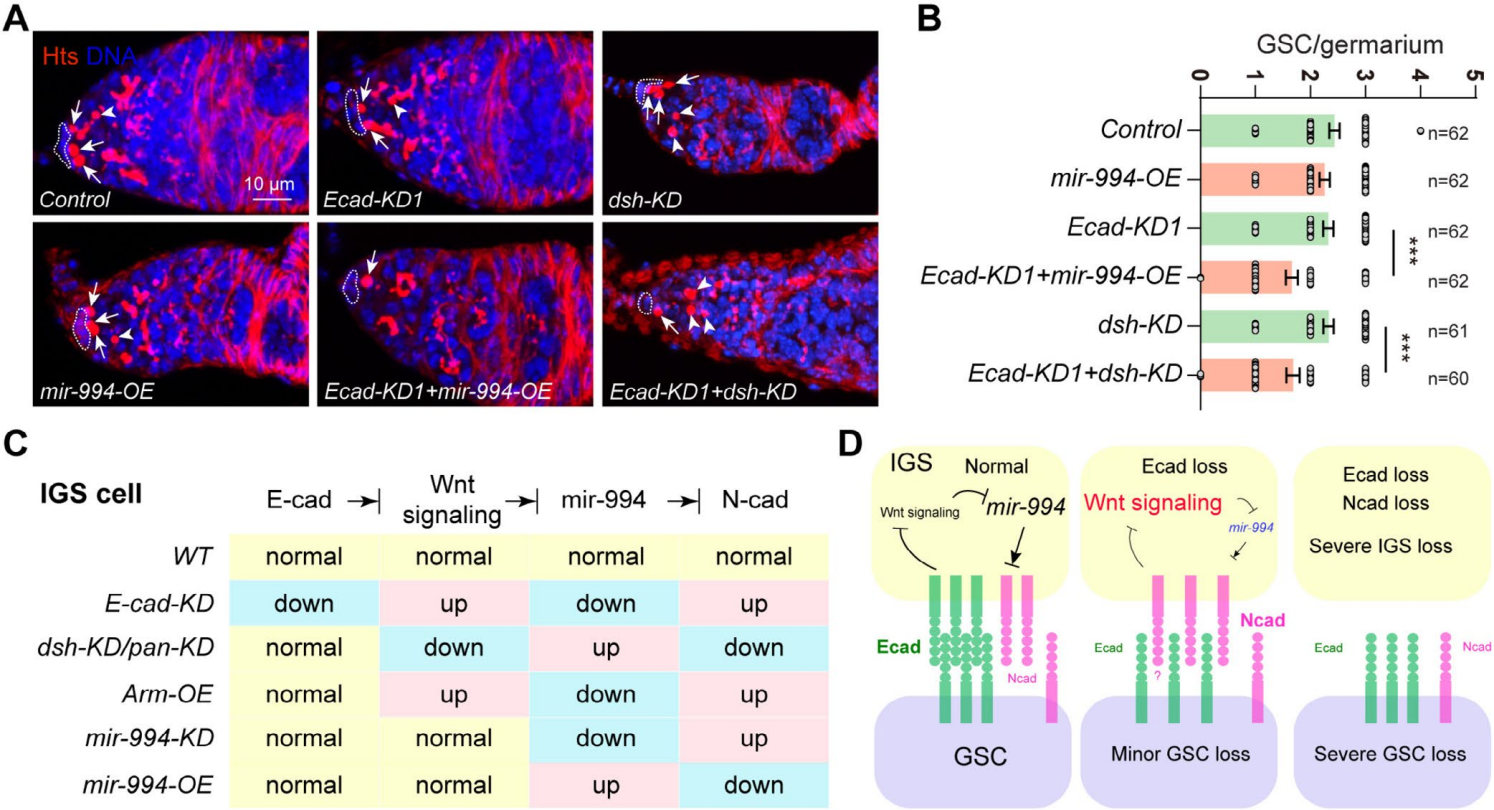
The Wnt signalling‐*mir‐994*‐mediated E‐cadherin‐to‐N‐cadherin switch in IGS cells controls GSC maintenance. (A, B) *mir‐994* overexpression (OE) alone did not affect the number of GSCs but *mir‐994‐OE* plus *Ecad‐KD* or *dsh‐KD* reduced the number of GSCs (*n* = number of germaria). Scale bars, 10 μm. (C) A proposed model explaining the expression relationship between Ecad, Wnt signalling, *mir‐994* and Ncad in IGS cells. (D) A working model illustrating how the Ecad‐Wnt signalling‐*mir‐994*‐Ncad axis might control the maintenance of IGS cells and GSCs. Student's *t*‐test: ***p ≤ 0.001.

Finally, we conducted genetic interaction tests to validate the functional relevance of the entire axis on GSC maintenance in vivo. First, we showed that overexpressing *mir‐994* in IGS cells did not significantly impact the GSCs when compared with the control (Figure 7A,B); this is consistent with the known unessential function of N‐cadherin in IGS cells. Second, we showed that knocking down E‐cadherin and overexpressing *mir‐994* in the same IGS cells resulted in a significant loss in the number of GSCs similar to the double knockdown of E‐ and N‐cadherins (compare Figure 7A,B with Figure 2A,B). Third, although *dsh‐KD* alone had no obvious impact on the number of GSCs, the *Ecad‐KD* and *dsh‐KD* double knockdown significantly decreased the GSC numbers by a similar amount as the E‐ and N‐cadherins double knockdown (compare Figure 7A,B with Figure 2A,B). These results conclusively demonstrate that the E‐cadherin‐Wnt‐*mir‐994*‐N‐cadherin regulatory axis functions as an integrated circuit to ensure the robust maintenance of GSCs (Figure 7C,D).

## 4 Discussion

### 4.1 A Repurposed Cadherin Switch Confers Robustness to the Stem Cell Niche

Stem cell niches provide a dedicated microenvironment to orchestrate stem cell behaviour, yet the mechanisms ensuring the stability and resilience of the niche itself are less well understood [6, 52]. Our study uncovers a novel regulatory circuit regarding cadherin switch and Wnt signalling in the *Drosophila* ovarian niche, which is repurposed to function as a fail‐safe mechanism, thereby ensuring the robustness of germline stem cell (GSC) maintenance. We have delineated a complete signalling axis, E‐cadherin‐Wnt‐*mir‐994*‐N‐cadherin, which controls the dynamic balance between these adhesion molecules in inner germarial sheath (IGS) cells, safeguarding niche function against fluctuations in E‐cadherin expression.

### 4.2 N‐cadherin Serves as a Context‐Dependent Compensator in the Niche

A pivotal finding of our work is the definition of N‐cadherin's role in IGS cells. While dispensable under homeostatic conditions, N‐cadherin becomes critically required upon the loss of E‐cadherin. This context‐dependent function resolves the apparent paradox of why the simultaneous depletion of both cadherins causes a catastrophic loss of IGS cells and GSCs, whereas neither single knockdown alone produces an equally severe phenotype. We demonstrate that the upregulated N‐cadherin compensates for E‐cadherin loss by maintaining IGS cell survival, proliferation and the integrity of their long cellular processes, which are essential for niche‐germ cell interactions. Notably, while posterior IGS cells do not directly contact GSCs, their altered adhesion profile may indirectly compromise niche function. We propose two non‐exclusive mechanisms: first, the disruption of cadherin‐based adhesion across the IGS cell network could impair the structural coordination and overall integrity of the niche, potentially affecting the supportive capacity of the anterior IGS cells that directly anchor GSCs. Second, changes in posterior IGS cells might perturb local signalling gradients or the physical niche architecture necessary for maintaining the stem cell microenvironment. This establishes N‐cadherin not as a redundant player, but as a dedicated backup system that is mobilized to preserve niche integrity during adhesion stress.

A previous paradigm suggests that IGS cell loss leads to germline differentiation defects [53]. While this holds true for developmental contexts, our adult‐specific knockdown model reveals a different outcome: the predominant consequence is GSC loss, which is likely caused by the timing and rate of IGS cell functional decay dictating the phenotypic output. This GSC loss likely stems from a combination of direct anchorage impairment in anterior IGS cells and the indirect effects on niche integrity initiated in posterior regions. Our data and previous studies suggest that the functional requirement of the niche can manifest as distinct phenotypes (differentiation arrest vs. stem cell loss) [10, 53].

### 4.3 A Non‐Canonical, Post‐Transcriptional Cadherin Switch Mechanism

The cadherin switch, wherein E‐cadherin loss leads to N‐cadherin gain, is a hallmark of EMT [54]. Although IGS cells are themselves non‐epithelial and non‐migratory, our findings demonstrate that the core molecular event of the cadherin switch—E‐cadherin loss leading to N‐cadherin upregulation—can be executed through a mechanism independent of the classical EMT program, reflecting a functional reuse of this molecular module in a non‐canonical context. E‐cadherin loss activates Wnt signalling, which in turn directly represses the transcription of *mir‐994*. This microRNA normally acts as a translational brake on N‐cadherin mRNA. Thus, the Wnt‐mediated suppression of *mir‐994* lifts this repression, leading to a robust upregulation of N‐cadherin protein without altering its mRNA levels. This E‐cadherin‐Wnt‐*mir‐994*‐N‐cadherin axis represents a previously unrecognized branch of cadherin cross‐regulation, highlighting the diversity of molecular strategies underlying this fundamental biological process.

### 4.4 The Partial Nature of N‐cadherin Compensation

Although our data support a model where N‐cadherin upregulation compensates for the loss of E‐cadherin, this compensation is ultimately partial. The significant decline in GSC numbers between 1 and 2 weeks of E‐cadherin knockdown indicates that N‐cadherin, while sufficient to transiently maintain niche integrity, cannot fully substitute for all essential functions of E‐cadherin in the long term. This functional divergence likely stems from the fundamental differences in their cytoplasmic binding partners and downstream signalling outputs, despite their shared ability to mediate homophilic adhesion [55, 56]. The upregulated N‐cadherin may effectively maintain general cell‐cell contact and prevent anoikis, thereby supporting IGS cell survival, but it fails to reconstitute the precise signalling microenvironment that E‐cadherin provides for sustained GSC self‐renewal. Therefore, the partial rescue we observe is a testament to a nuanced biological reality: cadherins possess both overlapping and unique functions. Our findings align with studies in other systems, such as the mammalian lens, where E‐ and N‐cadherin co‐expression is essential, yet their deletion yields distinct phenotypic outcomes, indicating non‐redundant roles.

### 4.5 Cadherin Switch Contributes to the Resilience of Stem Cell Niche

Our findings extend the understanding of the cadherin switch. In the context of the stem cell niche, this molecular event is uncoupled from its traditional role in promoting cell migration and invasion. Instead, it is harnessed to enforce stem cell‐niche interaction and niche resilience. The existence of a miRNA‐mediated, post‐transcriptional cadherin switch may allow for a more rapid and potentially reversible response to adhesion challenges, a feature that could be crucial for maintaining tissue homeostasis.

The broader implications of our work are underscored by the conservation of this regulatory network, as Wnt signalling, E‐ and N‐cadherin are evolutionarily conserved. Therefore, the regulatory axis we identified offers a new paradigm for understanding how stem cell niches maintain their functional integrity. It also presents a fresh perspective on diseases like cancer, where the hijacking of such a ‘robustness circuit’ could contribute to the resilience of cancer stem cell niches and their resistance to therapy.

## 5 Conclusion

Our study moves beyond establishing the mere requirement of adhesion molecules in the niche to revealing a sophisticated regulatory circuitry that ensures its stability. We have shown that the interplay between E‐ and N‐cadherin is not static but is dynamically controlled by a dedicated signalling pathway that can sense and compensate for adhesion deficits. By demonstrating that the molecular hardware of the cadherin switch is co‐opted to maintain—rather than disrupt—cellular architecture and function in the stem cell niche, our work provides a new conceptual framework for understanding niche robustness, with likely significant ramifications for developmental biology and cancer research.

## Author Contributions

R.T., H.L.Y. and R.D. conducted the experiments, and collected and analysed the data. S.C. conducted bioinformatics analysis. J.N. provided the necessary reagents. R.T. and T.X. designed the project and wrote the manuscript and S.E.W. helped prepare the manuscript.

## Funding

This work was supported by grants from the Research Grants Council of Hong Kong (TRS_T13‐602/21‐N, GRF_16104621 and GRF_16103822 to T.X.), National Natural Science Foundation of China (32400450 to R.T.) and Natural Science Foundation of Jiangsu Province (BK20241302 to R.T.).

## Ethics Statement

This study utilized the invertebrate model organism *Drosophila melanogaster*. As such, no specific ethics approval was required for this research.

## Conflicts of Interest

The authors declare no conflicts of interest.

## Supporting information


**Figure S1:** The localization of E‐cadherin (Ecad) and N‐cadherin (Ncad) in the *Drosophila* ovary. (A, B) Representative confocal fluorescence images to show the localization of Ecad (red) and nuclei (blue) (A) and Ncad (B). Ncad expression was observed in IGS cells (arrows). (C–F) *N‐cadherin* (*Ncad*) and *E‐cadherin* (*Ecad*) RNAi strains significantly knocked down the expression of Ncad (B, C) and Ecad (D, E), respectively, in cap cells driven by *bab1‐Gal4* ^*ts*^ (*n* = number of ROIs). Scale bars, 5 μm. (G) Fluorescence images show that the *c587* ^*ts*^‐mediated knockdown of *Ncad* almost entirely depleted the expression of N‐cad protein in the germaria (regions bounded by dotted white lines) excluding the cap cells. Student's *t*‐test: **p ≤ 0.001.
**Figure S2:** Germaria remain normal when genes are knocked down at the permissive temperature. (A, B) *c587* ^*ts*^‐mediated IGS‐specific knockdown of Ncad or Ecad alone, or the double knockdown of both (Double‐KD1) at 21°C showed normal numbers of GSCs and CBs such that no significant differences (n.s.) were observed among the data compared with the *luc* *‐* *KD* control (n = number of germaria). Scale bars, 10 μm. Student's t‐test: n.s., no significance.
**Figure S3:** N‐cadherin is dispensable for E‐cadherin expression in IGS cells. (A, B) Fluorescence images (A) and quantification results (B) show that the *c587* ^*ts*^‐mediated knockdown of Ncad has no effect on the expression of E‐cad protein in the IGS cells (n = number of ROIs). Scale bars, 10 μm (left) and 2 μm (right). Student's t‐test: n.s., no significance.
**Figure S4:** Overexpression of Ecad or Ncad partially rescues GSC loss caused by double knockdown in IGS cells. (A, B) *c587^ts^*‐mediated IGS‐specific overexpression of Ncad (2w) showed normal numbers of GSCs and CBs such that no significant differences were observed among the data when compared with the *UAS* *‐* *luc* overexpression control (n = number of germaria). Scale bars, 10 μm. (C, D) While IGS‐specific double knockdown of Ecad/Ncad (2w) induced GSC loss, concurrent overexpression of E‐cad partially rescued this phenotype. Simultaneous overexpression of both cadherins achieved better rescue efficiency. To control for potential competition between UAS elements for limited Gal4 protein, we included *UAS* *‐* *luc* *‐* *RNAi* (*luc* *‐* *KD*) or *UAS* *‐*luc in all experimental genotypes, ensuring an equal number of UAS transgenes across conditions (n = number of germaria). Scale bars, 10 μm. Student's t‐test: ***p ≤ 0.001, n.s., no significance.
**Figure S5:** The mRNA expression level of Ncad remained unchanged following Ecad knockdown in IGS cells. (A) Gene Ontology (GO) term enrichment analysis of differentially expressed genes. (B) RNA‐seq data revealed that the Ncad mRNA expression level was normal in Ecad‐KD IGS cells (n = number of biological replicates). (C, D) HCR‐FISH showed that the expression of *Ncad* mRNA was comparable in *luc* *‐* *KD* and *Ecad* *‐* *KD* IGS cells (Vasa‐negative, region bounded by the white line) (n = number of ROIs). Scale bars, 2 μm. Student's t‐test: n.s., no significance.
**Figure S6:** Altering *mir* *‐* *994* expression has no effect on *N* *‐* *cadherin* mRNA levels. (A) Volcano plot displaying pri‐miRNA expression upon IGS‐specific E‐cadherin knockdown. A total of 51 miRNAs were reliably detected. Two miRNAs were significantly downregulated (blue) and 0 miRNAs were significantly upregulated (red), compared to wild‐type controls (adjusted p‐value < 0.05). (B) Heatmap depicting the relative expression patterns of *mir*‐*994* and *mir* *‐* *986* in *E*‐*cadherin* RNAi and Control samples. (C, D) HCR‐FISH showing that *Ncad* mRNA expression is comparable in *luc* *‐* *KD*, *mir* *‐* *994*‐*KD*, and *mir* *‐* *994* *‐* *OE* IGS cells (Vasa‐negative as shown by region bounded by white line) (n = number of ROIs). Scale bars, 2 μm. Student's t‐test: n.s., no significance.
**Figure S7:** E‐ and N‐cadherin cooperate to sustain the long cellular processes of IGS cells. (A, B) By utilizing the FLP‐out system, individual IGS cells were labelled with GFP. Following *luc* *‐* *KD*, *Ncad* *‐* *KD*, or *Ecad* *‐* *KD*, the GFP‐labelled IGS cells still extended long cellular processes into the germarium, whereas following *Double* *‐* *KD*, the labelled IGS cell did not have long cellular processes (n = number of GFP‐labelled IGS cells). Scale bars, 10 μm.

**Table S1:** The raw data for all statistical graphs.

## Data Availability

The RNA‐seq data generated in this study have been deposited in the NCBI Gene Expression Omnibus (GEO) database under accession code GSE318190. All data supporting the findings of this study are provided in the text of this paper and related Supporting Information. All the original data and the reagents generated in this study are available upon request (Ting Xie: tgx@ust.hk and Renjun Tu: turenjun@seu.edu.cn).
