# Supplementary material for: The E‐cadherin‐Wnt‐mir‐994 Axis Repurposes a Cadherin Switch for Niche Robustness and Germline Stem Cell Maintenance
Source: Cell Prolif. 2026 Jun 26;59(11):e70245. doi: 10.1111/cpr.70245 (PMC13326017; doi:10.1111/cpr.70245)
Supplement: Supplementary file 1 [file CPR-59-e70245-s001.docx]

**FIGURE S1 |** The localization of E-cadherin (Ecad) and N-cadherin (Ncad) in the *Drosophila* ovary. **(A, B)** Representative confocal fluorescence images to show the localization of Ecad (red) and nuclei (blue) **(A)** and Ncad **(B)**. Ncad expression was observed in IGS cells (arrows). (**C-F**) *N-cadherin* (*Ncad*) and *E-cadherin* (*Ecad*) RNAi strains significantly knocked down the expression of Ncad (**B, C**) and Ecad (**D, E**), respectively, in cap cells driven by *bab1-Gal4^ts^* (n=number of ROIs). Scale bars, 5 µm. **(G)** Fluorescence images show that the *c587^ts^*-mediated knockdown of *Ncad* almost entirely depleted the expression of N-cad protein in the germaria (regions bounded by dotted white lines) excluding the cap cells. Student’s t-test: **p ≤ 0.001.

**FIGURE S2 |** Germaria remain normal when genes are knocked down at the permissive temperature. (**A, B**) *c587^ts^-*mediated IGS-specific knockdown of *Ncad* or *Ecad* alone, or the double knockdown of both (*Double-KD1)* at 21℃ showed normal numbers of GSCs and CBs such that no significant differences (n.s.) were observed among the data compared with the *luc-KD* control (n=number of germaria). Scale bars, 10 µm. Student’s t-test: n.s., no significance.

**FIGURE S3 |** N-cadherin is dispensable for E-cadherin expression in IGS cells. **(A, B)** Fluorescence images **(A)** and quantification results **(B)** show that the *c587^ts^*-mediated knockdown of *Ncad* has no effect on the expression of E-cad protein in the IGS cells (n=number of ROIs). Scale bars, 10 µm (left) and 2 µm (right). Student’s t-test: n.s., no significance.

**FIGURE S4 |** Overexpression of Ecad or Ncad partially rescues GSC loss caused by double knockdown in IGS cells. **(A, B)** *c587^ts^-*mediated IGS-specific overexpression of Ncad (2w) showed normal numbers of GSCs and CBs such that no significant differences were observed among the data when compared with the *UAS-luc* overexpression control (n=number of germaria). Scale bars, 10 µm. **(C, D)** While IGS-specific double knockdown of Ecad/Ncad (2w) induced GSC loss, concurrent overexpression of E-cad partially rescued this phenotype. Simultaneous overexpression of both cadherins achieved better rescue efficiency. To control for potential competition between UAS elements for limited Gal4 protein, we included *UAS-luc-RNAi (luc-KD)* or *UAS-luc* in all experimental genotypes, ensuring an equal number of UAS transgenes across conditions (n=number of germaria). Scale bars, 10 µm. Student’s t-test: ***p ≤ 0.001, n.s., no significance.

**FIGURE S5 |** The mRNA expression level of *Ncad* remained unchanged following *Ecad* knockdown in IGS cells. (A) Gene Ontology (GO) term enrichment analysis of differentially expressed genes. (**B**) RNA-seq data revealed that the *Ncad* mRNA expression level was normal in *Ecad-KD* IGS cells (n= number of biological replicates). (**C, D**) HCR-FISH showed that the expression of *Ncad* mRNA was comparable in *luc-KD* and *Ecad-KD* IGS cells (Vasa-negative, region bounded by the white line) (n=number of ROIs). Scale bars, 2 µm. Student’s t-test: n.s., no significance.

**FIGURE S6 |** Altering mir-994 expression has no effect on N-cadherin mRNA levels. (**A**) Volcano plot displaying pri-miRNA expression upon IGS-specific E-cadherin knockdown. A total of 51 miRNAs were reliably detected. Two miRNAs were significantly downregulated (blue) and 0 miRNAs were significantly upregulated (red), compared to wild-type controls (adjusted p-value < 0.05). (**B**) Heatmap depicting the relative expression patterns of *mir-994* and *mir-986* in E-cadherin RNAi and Control samples. (**C, D**) HCR-FISH showing that *Ncad* mRNA expression is comparable in *luc-KD, mir-994-KD, and mir-994-OE* IGS cells (Vasa-negative as shown by region bounded by white line) (n=number of ROIs). Scale bars, 2 µm. Student’s t-test: n.s., no significance.

**FIGURE S7 |** E- and N-cadherin cooperate to sustain the long cellular processes of IGS cells. (**A, B**) By utilizing the FLP-out system, individual IGS cells were labeled with GFP. Following *luc-KD*, *Ncad-KD*, or *Ecad-KD,* the GFP-labeled IGS cells still extended long cellular processes into the germarium, whereas following *Double-KD,* the labeled IGS cell did not have long cellular processes (n=number of GFP-labeled IGS cells). Scale bars, 10 µm.
