# Supplementary material for: The E‐cadherin‐Wnt‐mir‐994 Axis Repurposes a Cadherin Switch for Niche Robustness and Germline Stem Cell Maintenance
Source: Cell Prolif. 2026 Jun 26:e70245. Online ahead of print. doi: 10.1111/cpr.70245 (PMC13326017; doi:10.1111/cpr.70245)
Supplement: Supplementary file 1 — Figure S1: The localization of E‐cadherin (Ecad) and N‐cadherin (Ncad) in the Drosophila ovary. (A, B) Representative confocal fluorescence images to show the localization of Ecad (red) and nuclei (blue) (A) and Ncad (B). Ncad expression was observed in IGS cells (arrows). (C–F) N‐cadherin (Ncad) and E‐cadherin (Ecad) RNAi strains significantly knocked down the expression of Ncad (B, C) and Ecad (D, E), respectively, in cap cells driven by bab1‐Gal4 ts (n = number of ROIs). Scale bars, 5 μm. (G) Fluorescence images show that the c587 ts ‐mediated knockdown of Ncad almost entirely depleted the expression of N‐cad protein in the germaria (regions bounded by dotted white lines) excluding the cap cells. Student's t‐test: **p ≤ 0.001. Figure S2: Germaria remain normal when genes are knocked down at the permissive temperature. (A, B) c587 ts ‐mediated IGS‐specific knockdown of Ncad or Ecad alone, or the double knockdown of both (Double‐KD1) at 21°C showed normal numbers of GSCs and CBs such that no significant differences (n.s.) were observed among the data compared with the luc ‐ KD control (n = number of germaria). Scale bars, 10 μm. Student's t‐test: n.s., no significance. Figure S3: N‐cadherin is dispensable for E‐cadherin expression in IGS cells. (A, B) Fluorescence images (A) and quantification results (B) show that the c587 ts ‐mediated knockdown of Ncad has no effect on the expression of E‐cad protein in the IGS cells (n = number of ROIs). Scale bars, 10 μm (left) and 2 μm (right). Student's t‐test: n.s., no significance. Figure S4: Overexpression of Ecad or Ncad partially rescues GSC loss caused by double knockdown in IGS cells. (A, B) c587ts ‐mediated IGS‐specific overexpression of Ncad (2w) showed normal numbers of GSCs and CBs such that no significant differences were observed among the data when compared with the UAS ‐ luc overexpression control (n = number of germaria). Scale bars, 10 μm. (C, D) While IGS‐specific double knockdown of Ecad/Ncad (2w) induced G [file CPR-9999-e70245-s002.docx]

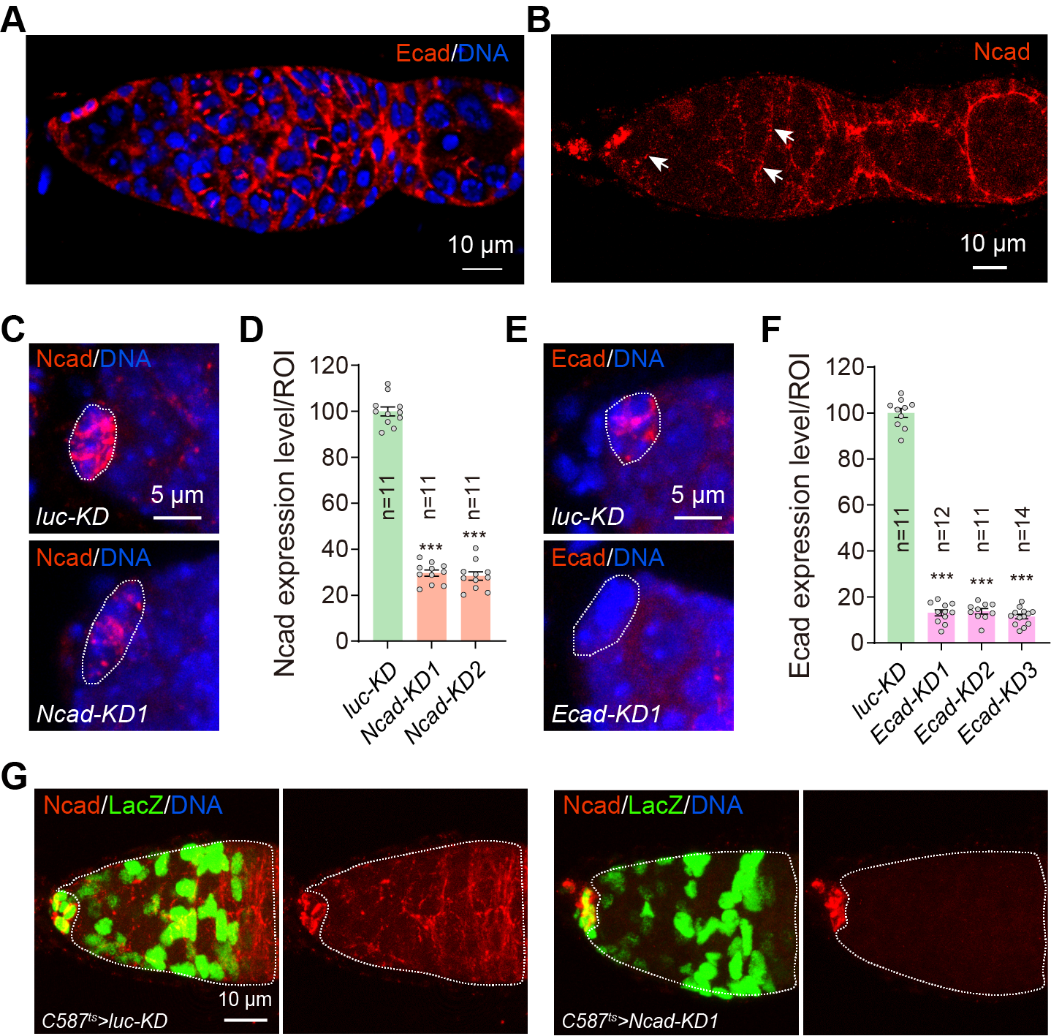


**FIGURE S1 |** The localization of E-cadherin (Ecad) and N-cadherin (Ncad) in the *Drosophila* ovary. **(A, B)** Representative confocal fluorescence images to show the localization of Ecad (red) and nuclei (blue) **(A)** and Ncad **(B)**. Ncad expression was observed in IGS cells (arrows). (**C-F**) *N-cadherin* (*Ncad*) and *E-cadherin* (*Ecad*) RNAi strains significantly knocked down the expression of Ncad (**B, C**) and Ecad (**D, E**), respectively, in cap cells driven by *bab1-Gal4^ts^* (n=number of ROIs). Scale bars, 5 µm. **(G)** Fluorescence images show that the *c587^ts^*-mediated knockdown of *Ncad* almost entirely depleted the expression of N-cad protein in the germaria (regions bounded by dotted white lines) excluding the cap cells. Student’s t-test: **p ≤ 0.001.


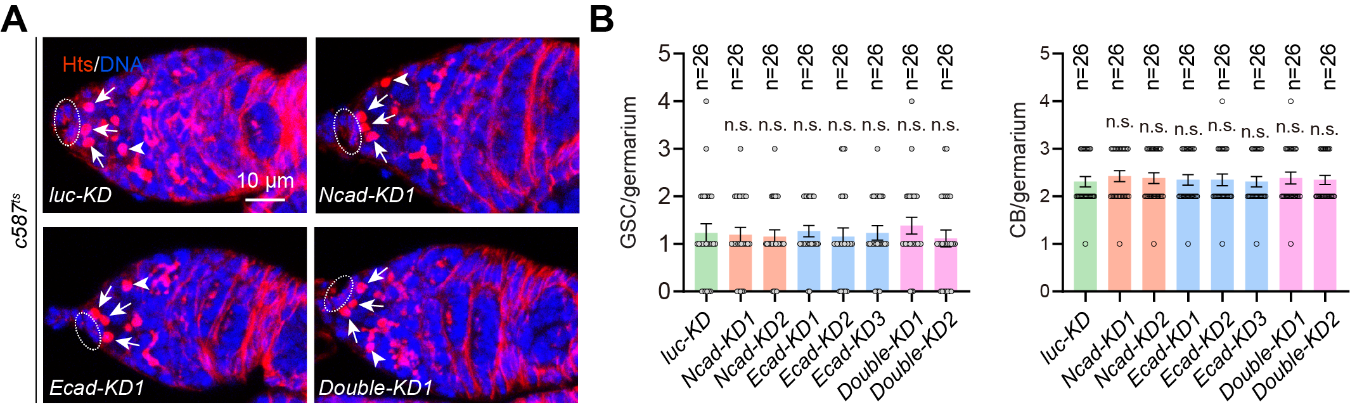


**FIGURE S2 |** Germaria remain normal when genes are knocked down at the permissive temperature. (**A, B**) *c587^ts^-*mediated IGS-specific knockdown of *Ncad* or *Ecad* alone, or the double knockdown of both (*Double-KD1)* at 21℃ showed normal numbers of GSCs and CBs such that no significant differences (n.s.) were observed among the data compared with the *luc-KD* control (n=number of germaria). Scale bars, 10 µm. Student’s t-test: n.s., no significance.


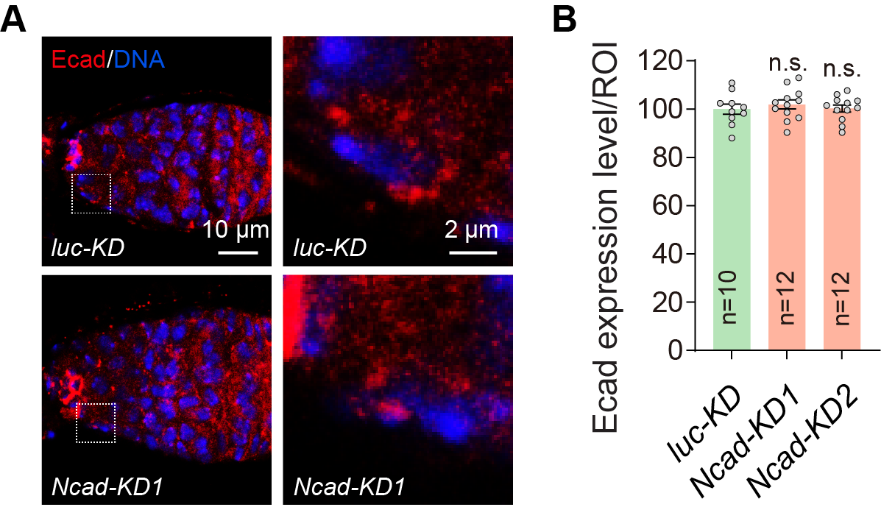


**FIGURE S3 |** N-cadherin is dispensable for E-cadherin expression in IGS cells. **(A, B)** Fluorescence images **(A)** and quantification results **(B)** show that the *c587^ts^*-mediated knockdown of *Ncad* has no effect on the expression of E-cad protein in the IGS cells (n=number of ROIs). Scale bars, 10 µm (left) and 2 µm (right). Student’s t-test: n.s., no significance.


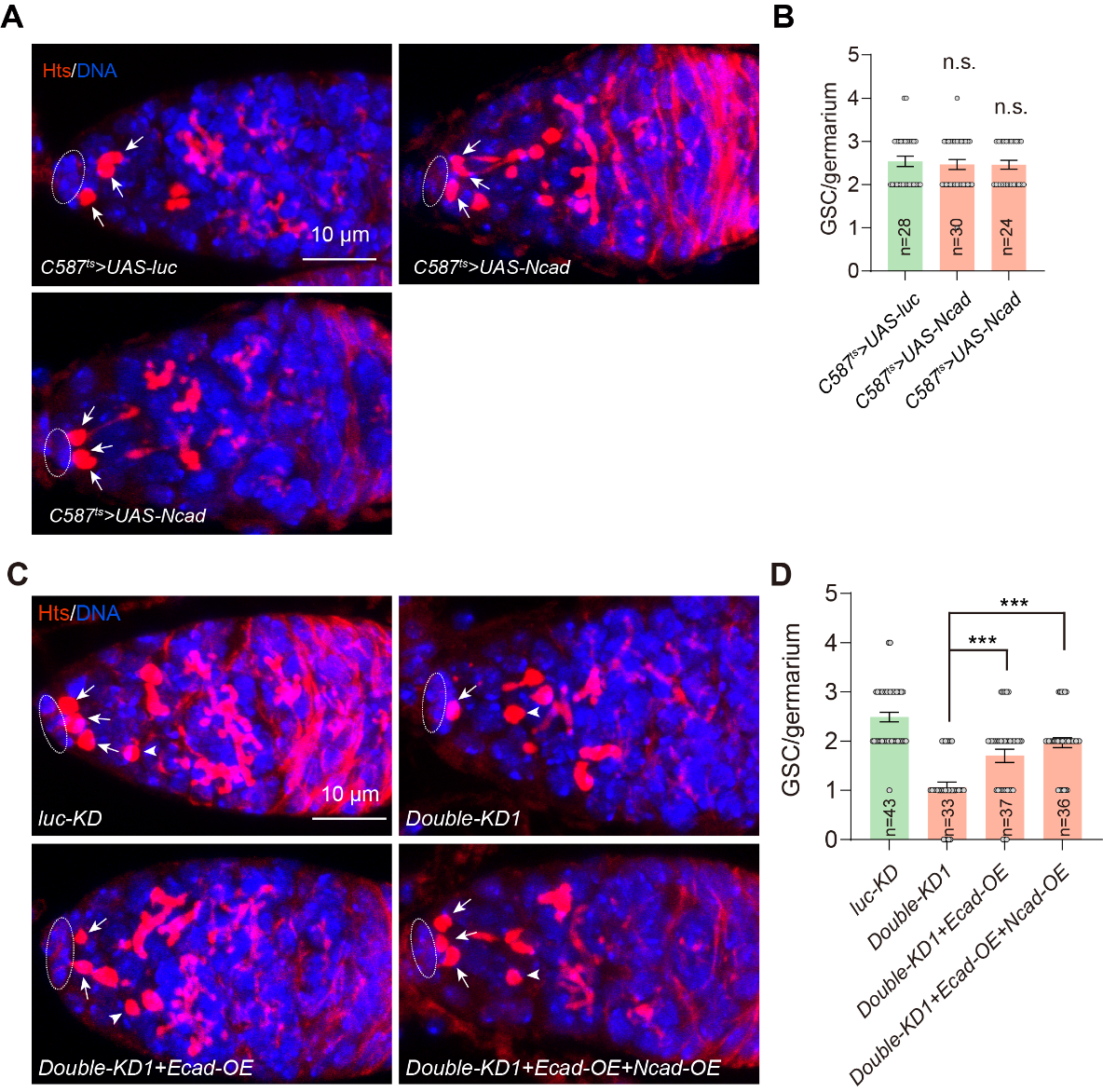


**FIGURE S4 |** Overexpression of Ecad or Ncad partially rescues GSC loss caused by double knockdown in IGS cells. **(A, B)** *c587^ts^-*mediated IGS-specific overexpression of Ncad (2w) showed normal numbers of GSCs and CBs such that no significant differences were observed among the data when compared with the *UAS-luc* overexpression control (n=number of germaria). Scale bars, 10 µm. **(C, D)** While IGS-specific double knockdown of Ecad/Ncad (2w) induced GSC loss, concurrent overexpression of E-cad partially rescued this phenotype. Simultaneous overexpression of both cadherins achieved better rescue efficiency. To control for potential competition between UAS elements for limited Gal4 protein, we included *UAS-luc-RNAi (luc-KD)* or *UAS-luc* in all experimental genotypes, ensuring an equal number of UAS transgenes across conditions (n=number of germaria). Scale bars, 10 µm. Student’s t-test: ***p ≤ 0.001, n.s., no significance.


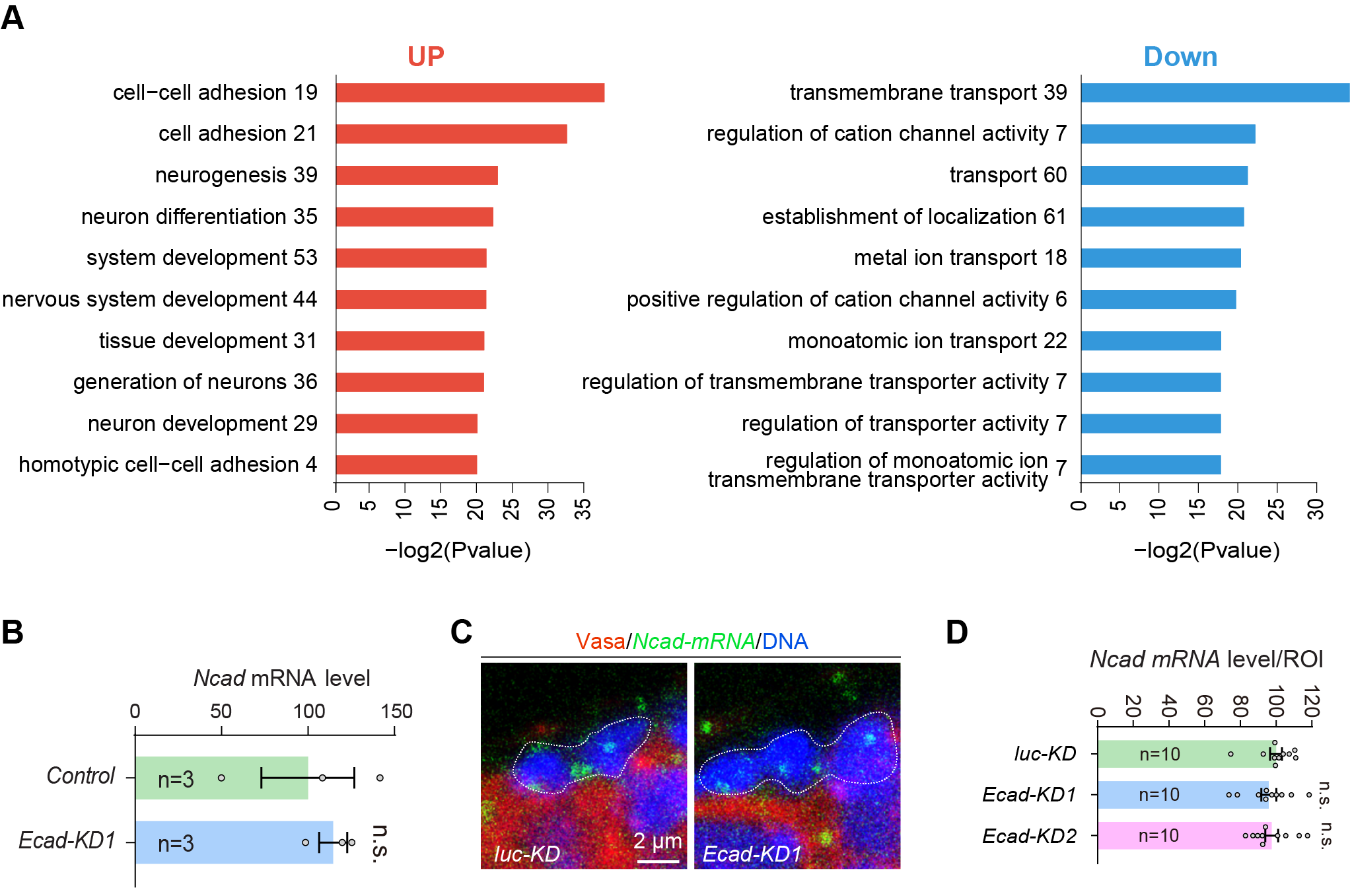


**FIGURE S5 |** The mRNA expression level of *Ncad* remained unchanged following *Ecad* knockdown in IGS cells. (A) Gene Ontology (GO) term enrichment analysis of differentially expressed genes. (**B**) RNA-seq data revealed that the *Ncad* mRNA expression level was normal in *Ecad-KD* IGS cells (n= number of biological replicates). (**C, D**) HCR-FISH showed that the expression of *Ncad* mRNA was comparable in *luc-KD* and *Ecad-KD* IGS cells (Vasa-negative, region bounded by the white line) (n=number of ROIs). Scale bars, 2 µm. Student’s t-test: n.s., no significance.


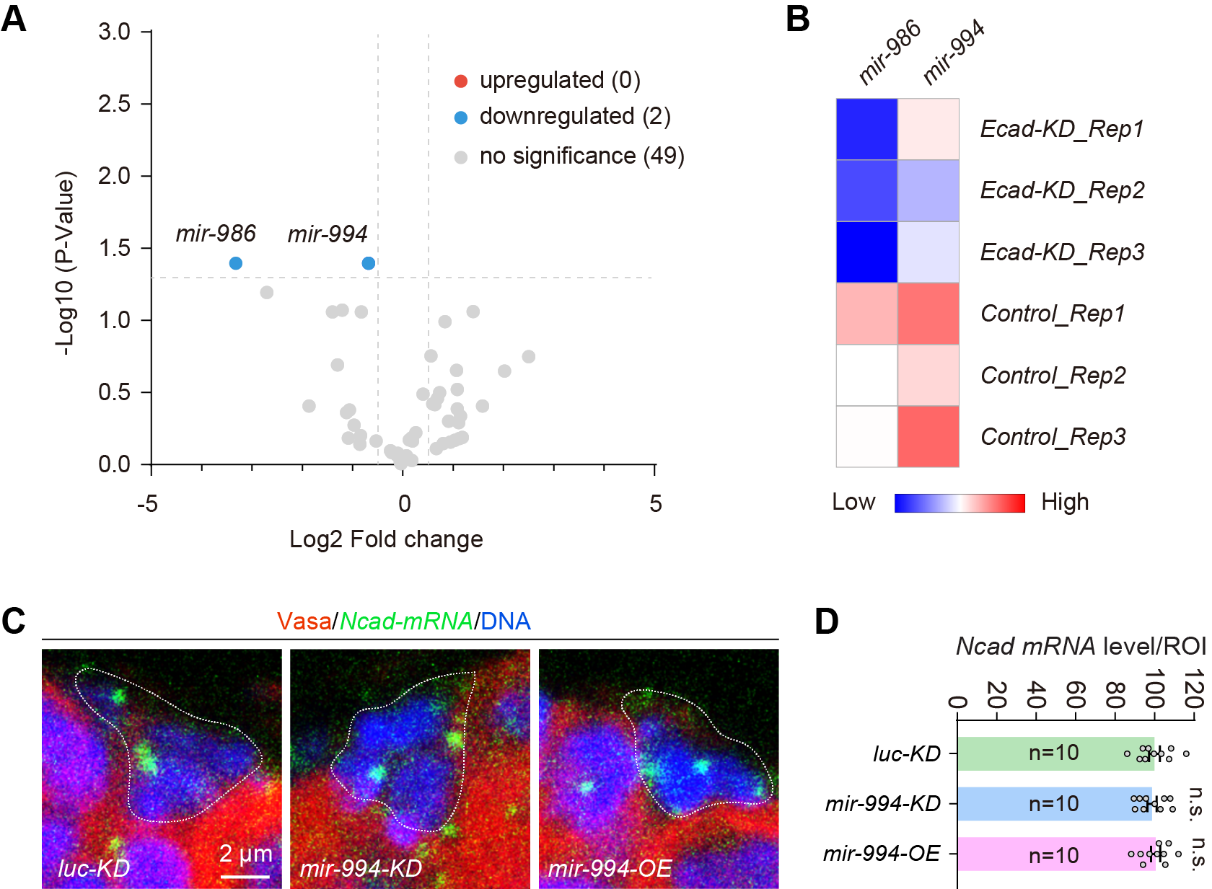


**FIGURE S6 |** Altering mir-994 expression has no effect on N-cadherin mRNA levels. (**A**) Volcano plot displaying pri-miRNA expression upon IGS-specific E-cadherin knockdown. A total of 51 miRNAs were reliably detected. Two miRNAs were significantly downregulated (blue) and 0 miRNAs were significantly upregulated (red), compared to wild-type controls (adjusted p-value < 0.05). (**B**) Heatmap depicting the relative expression patterns of *mir-994* and *mir-986* in E-cadherin RNAi and Control samples. (**C, D**) HCR-FISH showing that *Ncad* mRNA expression is comparable in *luc-KD, mir-994-KD, and mir-994-OE* IGS cells (Vasa-negative as shown by region bounded by white line) (n=number of ROIs). Scale bars, 2 µm. Student’s t-test: n.s., no significance.


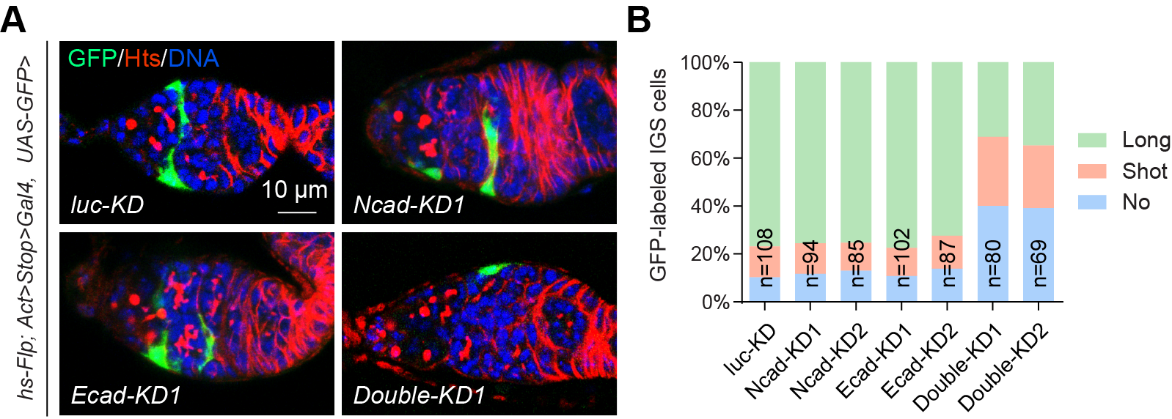


**FIGURE S7 |** E- and N-cadherin cooperate to sustain the long cellular processes of IGS cells. (**A, B**) By utilizing the FLP-out system, individual IGS cells were labeled with GFP. Following *luc-KD*, *Ncad-KD*, or *Ecad-KD,* the GFP-labeled IGS cells still extended long cellular processes into the germarium, whereas following *Double-KD,* the labeled IGS cell did not have long cellular processes (n=number of GFP-labeled IGS cells). Scale bars, 10 µm.
